# *Orientia tsutsugamushi* ankyrin repeat-containing protein family members are Type 1 secretion system substrates that traffic to the host cell endoplasmic reticulum

**DOI:** 10.3389/fcimb.2014.00186

**Published:** 2015-02-03

**Authors:** Lauren VieBrock, Sean M. Evans, Andrea R. Beyer, Charles L. Larson, Paul A. Beare, Hong Ge, Smita Singh, Kyle G. Rodino, Robert A. Heinzen, Allen L. Richards, Jason A. Carlyon

**Affiliations:** ^1^Department of Microbiology and Immunology, Virginia Commonwealth University School of MedicineRichmond, VA, USA; ^2^Coxiella Pathogenesis Section, Rocky Mountain Laboratories, National Institute of Allergy and Infectious Diseases, National Institutes of HealthHamilton, MT, USA; ^3^Viral and Rickettsial Diseases Department, Naval Medical Research CenterSilver Spring, MD, USA

**Keywords:** *Rickettsia*, intracellular bacteria, bacterial effector, scrub typhus, ankyrin repeat, bacterial secretion, ER-tropic effector, secretory pathway

## Abstract

Scrub typhus is an understudied, potentially fatal infection that threatens one billion persons in the Asia-Pacific region. How the causative obligate intracellular bacterium, *Orientia tsutsugamushi*, facilitates its intracellular survival and pathogenesis is poorly understood. Many intracellular bacterial pathogens utilize the Type 1 (T1SS) or Type 4 secretion system (T4SS) to translocate ankyrin repeat-containing proteins (Anks) that traffic to distinct subcellular locations and modulate host cell processes. The *O. tsutsugamushi* genome encodes one of the largest known bacterial Ank repertoires plus T1SS and T4SS components. Whether these potential virulence factors are expressed during infection, how the Anks are potentially secreted, and to where they localize in the host cell are not known. We determined that *O. tsutsugamushi* transcriptionally expresses 20 unique *ank* genes as well as genes for both T1SS and T4SS during infection of mammalian host cells. Examination of the Anks' C-termini revealed that the majority of them resemble T1SS substrates. *Escherichia coli* expressing a functional T1SS was able to secrete chimeric hemolysin proteins bearing the C-termini of 19 of 20 *O. tsutsugamushi* Anks in an HlyBD-dependent manner. Thus, *O. tsutsugamushi* Anks C-termini are T1SS-compatible. Conversely, *Coxiella burnetii* could not secrete heterologously expressed Anks in a T4SS-dependent manner. Analysis of the subcellular distribution patterns of 20 ectopically expressed Anks revealed that, while 6 remained cytosolic or trafficked to the nucleus, 14 localized to, and in some cases, altered the morphology of the endoplasmic reticulum. This study identifies *O. tsutsugamushi* Anks as T1SS substrates and indicates that many display a tropism for the host cell secretory pathway.

## Introduction

Scrub typhus is an acute, febrile, and potentially fatal zoonosis that is endemic to the Asia-Pacific region. One billion persons are at risk for the disease, and one million cases are estimated to occur annually. Scrub typhus can account for up to 20% of undifferentiated febrile hospitalizations in rural Asia, and unless treated with appropriate antibiotics, has an estimated 10% case fatality rate. Clinical manifestations include fever, rash, pneumonitis, disseminated vasculitis, systemic vascular collapse, and loss of cardiac function. The etiologic agent of scrub typhus is *Orientia tsutsugamushi*, a Gram-negative obligate intracellular bacterium of the Order *Rickettsiales* that is transmitted to humans during the feeding of the larval, or chigger, stage of infected trombiculid mites (Valbuena and Walker, 2012; Paris et al., 2013). The bacterium invades leukocytes at the chigger bite site. The infected leukocytes traffic to regional lymph nodes and disseminate to the peripheral vascular system. The pathogen ultimately egresses from leukocytes to infect endothelial cells of the skin and major organs (Paris et al., 2012, 2013). During the initial hours following uptake into host cells, the pathogen escapes its host cell-derived vacuole and replicates in the cytosol (Ge and Rikihisa, 2011). Considerable *O. tsutsugamushi* strain diversity exists (Valbuena and Walker, 2012; Paris et al., 2013), and the bacterial factors that facilitate survival in host cells are largely uncharacterized. Genomes of the Ikeda and Boryong strains, which were isolated from scrub typhus patients in Japan and Korea, respectively (Chang et al., 1990; Ohashi et al., 1996), have been sequenced and annotated (Cho et al., 2007; Nakayama et al., 2008). These genomes are valuable resources for investigations of *O. tsutsugamushi* molecular pathogenesis.

Ankyrin repeat-containing proteins (Anks) are key virulence factors of intracellular bacterial pathogens (Al-Khodor et al., 2010; Rikihisa and Lin, 2010; Voth, 2011; Jernigan and Bordenstein, 2014). These proteins contain one or more ankyrin repeats, each of which consists of a 33-residue motif that comprises the most common protein-protein interaction motif in nature. These domains form helix-turn-helix structures within proteins that mediate interactions with target proteins (Al-Khodor et al., 2010). Anks are capable of binding a diversity of targets for two reasons. First, the numbers of individual ankyrin repeats within Anks vary, which in turn affects their structure and flexibility as interaction platforms. Second, the high amino acid sequence degeneracy of each individual ankyrin repeat facilitates specificity of interactions (Jernigan and Bordenstein, 2014). Anks were first thought to be exclusive to eukaryotes and shown to be involved in a variety of processes including signal transduction, vesicular trafficking, cytoskeleton integrity, and transcriptional regulation (Al-Khodor et al., 2010; Voth, 2011; Jernigan and Bordenstein, 2014). However, many intracellular bacterial species translocate Ank effectors into host cells, which traffic to distinct subcellular locales and subvert eukaryotic processes beneficial to the pathogens (Park et al., 2004; Ijdo et al., 2007; Lin et al., 2007; Pan et al., 2008; Garcia-Garcia et al., 2009; Price et al., 2009, 2010a,b, 2011; Voth et al., 2009; Lomma et al., 2010; Luhrmann et al., 2010; Mukherjee et al., 2011; Campanacci et al., 2013).

A recent assessment of 1912 bacterial genomes revealed that 51% of them encode at least one Ank, evidencing their widespread distribution among prokaryotes. The same study also concluded that the exceptionally high composition of Anks in the proteomes of obligate intracellular bacteria surpasses that of other bacterial lifestyles and is comparable to the composition of Anks of eukaryotes (Jernigan and Bordenstein, 2014). The *O. tsutsugamushi* Ikeda strain genome carries 47 Ank open reading frames (ORFs) (Nakayama et al., 2008), one of the highest numbers of any obligate intracellular bacterium (Jernigan and Bordenstein, 2014). Maintenance of such a large repertoire of Ank ORFs over the course of its reductive evolution as an obligate intracellular organism implies the importance of the Anks to *O. tsutsugamushi* pathobiology.

To exert their modulatory effects, Anks must be translocated from bacteria into host cells. Several *Rickettsiales* effectors, including Anks, are deployed by the Type 1 or Type 4 secretion system (T1SS or T4SS) (Lin et al., 2007; Huang et al., 2010; Wakeel et al., 2011; Kaur et al., 2012). The T1SS translocates proteins from the cytoplasm of Gram-negative bacterial pathogens to the extracellular milieu in a single step. The T1SS consists of three cell envelope proteins, each of which is required for secretion: an ATP-binding cassette (ABC) transporter localized in the inner membrane that recognizes substrates and ensures specificity of the translocation process; a membrane fusion protein (MFP) that is a periplasmic adaptor linking the cytoplasmic and outer membrane components; and an outer membrane protein (OMP) that forms a channel that remains closed until substrate recognition. Interaction of a T1SS substrate's secretion signal with the ABC triggers assembly of the apparatus and a conformational change in the OMP to yield the functional T1SS apparatus (Thomas et al., 2014).

Bacterial T1SS effectors have been shown to be toxins, proteases, lipases, heme-binding proteins, adhesins, S-layer proteins, or modulators of host cell factors involved in critical eukaryotic pathways (Zhu et al., 2009; Luo et al., 2011; Wakeel et al., 2011; Luo and Mcbride, 2012; Thomas et al., 2014). While there is no consensus T1SS substrate signal sequence, it typically comprises the C-terminal 60 residues, is rich in several amino acids (LDAVTSIF), poor in others (KHPMWC), and is not cleaved during translocation (Delepelaire, 2004; Wakeel et al., 2011; Thomas et al., 2014). Additionally, T1SS effectors tend to have acidic pIs and contain few or no cysteine residues.

The most studied T1SS is the *Escherichia coli* hemolysin A (HlyA) secretion system. Recognition of the HlyA C-terminal secretion signal by HlyB (ABC transporter) triggers recruitment of HlyD (MFP) and TolC (OMP) to the membrane, followed by T1SS assembly and HlyA secretion (Thomas et al., 2014). The HlyA secretion system is a valuable tool for studying the T1SS effectors of other bacterial species, especially those that are genetically intractable. It will secrete heterologous T1SS substrates of the *Rickettsiales* members *Ehrlichia chaffeensis* (Wakeel et al., 2011) and *Rickettsia typhi* (Kaur et al., 2012), as well as other bacteria (Masure et al., 1990; Thompson and Sparling, 1993), when expressed in *E. coli*. The HlyA T1SS also translocates recombinant HlyA chimeric proteins in which the HlyA C-terminal secretion signal has been replaced by secretion signals of heterologous T1SS substrates (Zhang et al., 1993, 1995).

The T4SS is another substrate translocation channel that traverses Gram-negative bacterial cell walls (Christie et al., 2014). T4SS effectors of several important human pathogens including *Coxiella burnetii, Legionella pneumophila, Helicobacter pylori, Brucella* spp., and *Anaplasma phagocytophilum* have been shown to modulate a wide range of host cell processes (Bhatty et al., 2013). The archetypal *Agrobacterium tumefaciens* VirB/VirD4 T4SS consists of 11 VirB proteins and VirD4. T4SSs of many other Gram-negative bacterial species consist of homologs of most or all *A. tumefaciens* VirB subunits and VirD4 (Christie et al., 2014). The *O. tsutsugamushi* genome encodes homologs of *A. tumefaciens* VirB1, VirB2, VirB3, VirB4, multiple near-identical copies of VirB6, VirB8, VirB9, VirB10, and VirD4 (Cho et al., 2007; Nakayama et al., 2008). Another T4SS that is distinct from the VirB/VirD4 apparatus is the Dot/Icm system that is carried by various Gram-negative pathogens, including *L. pneumophila* and *C. burnetii*. The VirB/VirD4 and Dot/Icm T4SSs have some discernible sequence homologies to each other (Bhatty et al., 2013). They retain at least some degree of functional homology because surrogate bacterial hosts that use the Dot/Icm system can secrete VirB/VirD4 substrates (De Jong et al., 2008; Huang et al., 2010). For both VirB/VirD4 and Dot/Icm substrates, the translocation signals tend to be located in their unstructured C-termini and can include clusters of positively charged arginine residues (VirB/D4), glutamate-rich stretches called E blocks (Dot/Icm), and hydrophobic residues (Dot/Icm) (Nagai et al., 2005; Vergunst et al., 2005; Huang et al., 2011).

Given the paucity of information on *O. tsutsugamushi* effectors, the importance of the T1SS and T4SS to rickettsial pathogens, and the potential of Anks to serve as virulence factors, we investigated the secretion mechanism of *O. tsutsugamushi* Ikeda strain Anks. *C. burnetii* could not secrete heterologously expressed Anks in a T4SS-dependent manner, but *E. coli* secreted chimeric HlyA proteins bearing the C-termini of *O. tsutsugamushi* Anks in an HlyBD-dependent manner. As a first step in elucidating their pathobiological roles, we assessed the subcellular locales to where the Anks trafficked when they were ectopically expressed in mammalian cells. Strikingly, 14 out of the 20 Anks examined localized to the endoplasmic reticulum (ER). This study provides the first experimental evidence for how any *O. tsutsugamushi* effector can be translocated and demonstrates the potential that a large portion of the *Orientia* Ank armamentarium has for disrupting the host cell secretory pathway.

## Materials and methods

### Cultivation of uninfected and *O. tsutsugamushi* infected host cells

L929 mouse fibroblast cells (CCL 1 NCTC Clone 929; American Type Culture Collection [ATCC], Manassas, VA) were grown in Eagle's Minimum Essential Medium (EMEM; Quality Biological, Gaithersburg, MD) supplemented with 2.5% (vol/vol) fetal bovine serum (FBS; Gemini Bio-Products, West Sacramento, CA) at 35°C in a humidified incubator with 5% CO_2._ For infecting cells with *O. tsutsugamushi*, L929 cells were γ-irradiated with 1500 rads followed by the addition of *O. tsutsugamushi* at a multiplicity of infection (MOI) of 10. Following the initial infection, the infected L929 cells were maintained as described for uninfected host cells. HeLa human cervical epithelial cells (CCL-2; ATCC) were maintained in Roswell Park Memorial Institute (RPMI)-1640 (Gibco, Grand Island, NY) medium supplemented with 10% FBS at 37°C in a humidified incubator with 5% CO_2_.

### *C. burnetii* cultivation and infection

*C. burnetii* Nine Mile RSA439 (phase II, clone 4) was cultivated axenically in ACCM-2 as previously described (Omsland et al., 2011). THP-1 human monocytic leukemia cells (TIB-202; ATCC) were maintained in RPMI-1640 medium containing 10% FBS at 37°C and 5% CO_2_. THP-1 monocytes were differentiated into macrophage-like cells by overnight treatment with 200 nM phorbol-12-myristate-13-acetate (PMA) followed by two washes with phosphate-buffered saline (PBS; 1.05 mM KH_2_PO_4_, 155 mM NaCl, 2.96 mM Na_2_HPO_4_, pH 7.2) prior to infection.

### *In silico* analyses of *O. tsutsugamushi* Anks

The Protein BLAST (Basic Local Alignment Search Tool) algorithm (http://blast.ncbi.nlm.nih.gov/Blast.cgi) (Altschul et al., 1990) was used to search each *O. tsutsugamushi* Ank sequence against the non-redundant protein sequences database. Identification of the amino acid locations of individual ankyrin repeats within each Ank was accomplished using the Simple Modular Architecture Research Tool (SMART) algorithm (http://smart.embl-heidelberg.de/) (Schultz et al., 1998; Ponting et al., 1999). The final C-terminal 60 amino acids of each Ank were visually screened for the presence of amino acids that are commonly found in the secretion signal sequences of T1SS effectors (LDAVTSIF) as well as those that are commonly absent (KHPMWC) (Delepelaire, 2004; Wakeel et al., 2011). The pI of each protein of interest and its C-terminus was calculated using the protein calculator algorithm (http://protcalc.sourceforge.net/).

### RNA isolation and RT-PCR

Total RNA from uninfected or *O. tsutsugamushi* infected cells (≥90% of host cells were infected; 11 days post infection) was isolated using the RNAqueous Total RNA Isolation kit (Life Technologies, Grand Island, NY) followed by treatment with RNAse-free DNAse (11.5 U/μg) (Invitrogen, Carlsbad, CA) for 1 h at 37°C. cDNA stocks (20 μl) were prepared from 1 μg of DNA-free RNA using random hexamers and the iScript cDNA Synthesis kit (Biorad, Hercules, CA) followed by PCR amplification using 2 μl of cDNA template and *O. tsutsugamushi* Ank gene-specific primers (Table A1). The thermal cycling conditions used were 94°C for 2 min, followed by 35 cycles of 94°C for 60 s, 55°C for 60 s, and 72°C for 60 s, with a final extension step of 72°C for 7 min. To ensure that RNA templates were free from contaminating DNA, identical RT-PCR reactions were performed in the absence of reverse transcriptase using primers specific for the *O. tsutsugamushi* 16S rRNA gene (OTT_RNA006) and murine ß-actin (data not shown). Representative amplicons generated using primers specific for *ank5_01, ank9, ank11, ank13*, and *ank17* were purified using the PCRExtract Mini Kit (5 Prime, Gaithersburg, MD) and sequenced (Genewiz, South Plainfield, NJ) to verify amplicon identity.

### Plasmids for subcellular localization studies and TISS assays

pBMH constructs carrying *O. tsutsugamushi ank* genes that were codon optimized for expression in mammalian cells were synthesized by Biomatik (Wilmington, DE; Table A2) with an *EcoRI* restriction site and a single nucleotide that restored the reading frame upstream of the gene and a *SalI* restriction site downstream of the gene. pBMH vectors containing the *ank* genes and the recipient vectors, p3XFLAG-CMV-7.1 (Sigma-Aldrich, St. Louis, MO) and pEGFP-C1 (donated by Marci Scidmore, Cornell University, Ithaca, NY), were double-digested with 0.8 U each of *SalI* and *EcoRI* (New England Biolabs, Ipswich, MA) for 2 h at 37°C in *EcoRI* buffer (New England Biolabs), and gel-purified using the QIAquick Gel Extraction Kit (Qiagen, Valencia, CA). The restriction fragments carrying each codon-optimized *ank* were ligated into the digested plasmids with T4 DNA ligase (New England Biolabs) according to the manufacturer's instructions. Biomatik synthesized *ank10_01* and *ank14* as mammalian codon-optimized sequences and cloned them into pEGFP-C1 to generate pEGFP-Ank10 and pEGFP-Ank14, respectively.

Because p3XFLAG-CMV-7.1 and pEGFP-C1 recombinant constructs carrying the *ank* genes expressed each Ank as an N-terminal fusion (Table A2), we also expressed representative Anks as C-terminal 3XFLAG-tag fusions to verify that placement of the fusion tag had no bearing on the subcellular localization of ectopically expressed Anks. To this end, we PCR amplified mammalian codon-optimized *ank4, ank9*, and *ank17* using gene-specific primers that also carried a *KpnI* site at the 5′ end of the forward primer or a *XbaI* site at the 5′ end of the reverse primer (Table A3). PCR was performed using Platinum Taq HiFi DNA Polymerase (Invitrogen) and the following thermal cycling conditions: 94°C for 2 min; 34 cycles of 94°C for 30 s, 55°C for 30 s, and 68°C for 2 min; followed by 68° for 6 min. The PCR products were purified using the PCR Clean-up kit (5 Prime) following the manufacturer's protocol. The p3XFLAG-CMV-14 recipient vector (Sigma-Aldrich) and the *ank* PCR products were each double-digested with 0.4 U of *KpnI* and 0.8 U of *XbaI* (New England Biolabs) for 2 h at 37°C, gel-purified, and ligated as described above.

To generate constructs for expressing N-terminal His-tagged chimeric HlyA proteins in which the final 60 C-terminal amino acids of HlyA were replaced with the final 60 C-terminal amino acids of each *O. tsutsugamushi* Ank, we utilized the In-Fusion HD Cloning Plus kit (Clontech, Mountain View, CA) following the manufacturer's protocol to PCR amplify and clone *hlyA-ank* fusion PCR products into the pET19-b vector (Novagen, Madison, WI). Primers for this purpose were designed using Clontech's online Primer Design Tool for In-Fusion Cloning (www.clontech.com) (Table A4). Each mammalian codon-optimized *ank* was PCR amplified from its respective pBMH construct (Table A2) using an *ank* gene-specific forward primer and a reverse primer that targeted the 3′ end of its respective *ank* and had an additional 17-nucleotide sequence at its 5′ end that was complementary to the insertion site of the pET19-b vector. *E. coli* strain A0 34/86 was kindly provided by Peter Sebo (Institute of Microbiology, Institute of Biotechnology, Academy of Sciences of the Czech Republic, Prague, Czech Republic). The *hlyA* gene minus its final 180 nucleotides and stop codon was PCR amplified from DNA that had been isolated from a boiled colony of *E. coli* strain A0 34/86 using *hlyA*-specific forward and reverse primers that targeted nucleotides 4 to 2892 of *hlyA* and had an additional 15-nucleotides at its 5′ end that were complementary to the first 15 nucleotides of the coding sequence for each Ank's putative Type 1 secretion signal (Table A4). PCR reactions were performed using CloneAmp HiFi PCR Premix (Clontech) and the following thermal cycling conditions: 98°C for 2 min; followed by 35 cycles of 98°C for 10 s, 55°C for 15 s, and 72°C for 60 s. PCR products were resolved by gel electrophoresis, excised, purified using the QIAquick Gel Extraction Kit (Qiagen), eluted, and assayed spectrophotometrically for DNA concentration. The pET19-b vector was digested with 0.4 U of *Nde*I at 37°C for 1 h and purified using the PCR Extract Mini Kit (5 Prime). The InFusion cloning reaction was performed according to the manufacturer's protocol and the resulting recombinant constructs were transformed into Stellar *E. coli* cells (Clontech). The transformants were plated on Luria-Bertani (LB) agar plates containing 100 μg/ml ampicillin and incubated at 37°C overnight. Colonies were screened via colony PCR using the *hlyA*-1731F forward primer (Table A4) and T7 terminator reverse primer (Integrated DNA Technologies, Coralville, IA), MyTaq Red polymerase and buffer (Bioline, Taunton, MA), and thermal cycling conditions described above. All constructs described in this section were isolated using the PerfectPrep Spin Mini Kit (5 Prime) and sequenced to verify insert integrity (Genewiz). The resulting plasmids that encode each HlyA-Ank chimeric protein consisting of HlyA lacking its final C-terminal 60 amino acids fused to the final 60 amino acids of each respective *O. tsutsugamushi* Ank are listed in Table A5.

### CyaA translocation assay

Mammalian codon-optimized Ank coding sequences were amplified from the plasmids listed in Table A2 using Accuprime Pfx (Invitrogen) and the oligonucleotides listed in Table A6. The PCR products were cloned into pJB-CAT-CyaA (Voth et al., 2011) using the In-Fusion HD Cloning Plus kit (Clontech) to generate plasmids encoding each Ank N-terminally fused to CyaA (Table A7). CyaA assays were conducted as previously described (Larson et al., 2013). Briefly, PMA-differentiated THP-1 macrophages seeded in 24-well plates at a density of 5 × 10^5^ cells per well were infected at a MOI of 50 with *C. burnetii* transformants expressing each candidate protein N-terminally fused to CyaA. After 48 h, the cells were washed with PBS and lysed with 200 μl of lysis buffer containing 50 mM HCl and 0.1% Triton X-100. Samples were boiled for 5 min and 400 μl of 95% ethanol was added. The samples were dried under vacuum and resuspended in 400 μl of assay buffer (0.5 M sodium acetate [pH 6.0], 0.002% w/v bovine serum albumin). The amount of cAMP in the samples was determined with the cAMP Biotrak Enzymeimmunoassay (EIA) System (GE Healthcare, Piscataway, NJ) according to the non-acetylation procedure. Samples were measured in duplicate for each of three independent experiments. Values were reported as fold change in cAMP concentration vs. the empty vector control (CyaA only). Proteins were deemed T4SS substrates if fold change in cAMP concentration was significantly greater (*P* > 0.05) than the CyaA only control using the One-Way ANOVA statistical test, part of the Prism 6.0 software package (GraphPad, La Jolla, CA).

### T1SS assay and western blot analyses

Plasmid pLG575 (Mackman et al., 1985), which constitutively expresses *E. coli* HlyB and HlyD, was kindly provided by Peter Sebo (Institute of Microbiology, Institute of Biotechnology, Academy of Sciences of the Czech Republic). On day one, *E. coli* BL21 (DE3) cells (Bioline) were transformed with pET19-b plasmids carrying inserts that encoded His-tagged HlyA-Ank fusion proteins (Table A5) in the presence or absence of pLG575. Transformants were cultivated overnight in LB broth containing the appropriate antibiotics at 37°C with shaking at 250 rpm to generate starter cultures. On day two, 1 ml of each culture was diluted twenty-fold into fresh media and appropriate antibiotics, and the cultures were shaken at 250 rpm at 37°C. When the OD was between 0.6 and 1.0, 0.5 ml of each culture was retained as a non-induced control. The cultures were chilled on ice for 10 min, after which they were induced to express proteins of interest by the addition of isopropyl ß-D-1-thiogalactopyranoside (IPTG) to a final concentration of 1.0 mM and shaking at 250 rpm at 16°C overnight. On day three, a 0.5 ml aliquot of each induced culture was removed, its OD determined to ensure that the *E. coli* had not lysed due to HlyA-Ank expression and for subsequent normalization when loading on a SDS-PAGE gel. Aliquots were pelleted at 11,000× g for 10 min, and the supernatant discarded. This saved bacterial pellet would enable us to verify that proteins of interest had been expressed. For supernatant samples, each culture was spun at 10,000× g for 20 min at 4°C, after which 15 ml of the supernatant was syringe filter-sterilized through a 0.22 μm filter, mixed with 15 ml of ice-cold 40% (vol/vol) trichloroacetic acid (TCA) in acetone, and chilled at 4°C for 1 h. The samples were spun at 13,000× g for 15 min, the supernatant discarded, and the pellets, which contained precipitated proteins, were each resuspended in 1 ml of ice-cold acetone and transferred to a 1.5 ml tube. The sample was spun at 15,000× g for 5 min at 4°C and washed with ice-cold acetone two additional times. After pipetting to remove most of the acetone, the tube containing precipitated proteins was heated at 70°C for 5 min to evaporate residual acetone. Precipitated proteins were resuspended in 25 μl of PBS.

Normalized amounts of the induced *E. coli* pellet samples and the entirety of the supernatant samples were resolved by SDS-PAGE in 4 to 15% polyacrylamide gradient gels (Biorad) at 115 V for 10 min followed by 200 V for 20 min. Proteins from the gels were transferred to nitrocellulose membrane in Towbin buffer at 100 V for 30 min. The blots were blocked in 5% (vol/vol) non-fat dry milk in tris-buffered saline plus 0.05% Tween-20 (TBS-T) for 1 h at room temperature. The blots were screened with mouse monoclonal antibody G3 that was specific for HlyA residues 626–673 (Rowe et al., 1994) (a kind gift from Rodney Welch, University of Wisconsin, Madison, WI) at a 1:5000 dilution in 1% (vol/vol) non-fat dry milk in TBS-T overnight at 4°C with gentle rocking. The blots were washed three times in TBS-T with vigorous agitation. They were incubated with horseradish peroxidase-conjugated horse anti-mouse IgG (Cell Signaling Technology, Danvers, MA) at a 1:10,000 dilution in 1% (vol/vol) non-fat dry milk in TBS-T for 1 h with rocking followed by five washes in TBS-T. Blots of *E. coli* pellets and supernatants were incubated with SuperSignal West Pico or SuperSignal West Dura (Thermo Scientific, Rockford, IL), respectively, and exposed to film.

### Ectopic expression and subcellular localization studies of *O. tsutsugamushi* Anks in mammalian host cells

HeLa cells were seeded onto glass coverslips in 24-well plates and transfected with 0.4 μg of plasmids expressing GFP- or Flag-tagged Anks using Lipofectamine 2000 (Invitrogen) as directed by manufacturer. At 18–24 h post transfection, cells were fixed in 4% (vol/vol) paraformaldehyde (PFA) (Electron Microscopy Science, Hatfield, PA) in PBS. Coverslips of cells expressing Flag-tagged proteins were blocked for 1 h at room temperature in PBS containing 5% (vol/vol) bovine serum albumin (BSA). Next, the coverslips were stained with mouse Flag antibody (Sigma-Aldrich) at a 1:1000 dilution for 1 h followed by Alexa Fluor 488-conjugated goat anti-mouse IgG antibody (Invitrogen) at a dilution of 1:1000 for 1 h. In some cases, fixed coverslips of cells ectopically expressing tagged Ank proteins were screened with chicken anti-GFP (Invitrogen), rabbit anti-calnexin (Enzo Life Sciences, Farmingdale, NY), and/or rabbit anti-calreticulin (Sigma-Aldrich) at dilutions recommended by the manufacturers. Chicken anti-GFP was detected using Alexa Fluor 488-conjugated goat anti-chicken IgG (Invitrogen) at a 1:1000 dilution. Alexa Fluor 594-conjugated goat anti-rabbit IgG (Invitrogen) diluted 1:1000 was used to detect rabbit primary antibodies. Primary and secondary antibodies were diluted in PBS containing 1% (vol/vol) BSA. Coverslips were mounted using ProLong Gold antifade plus 4′,6-diamidino-2-phenylindole (DAPI) (Invitrogen) and imaged with a Zeiss LSM 700 laser-scanning confocal microscope.

### Generation of Ank4 antiserum and validation of its specificity using a Flag-Ank4 immunoprecipitation assay

Affinity purified rabbit polyclonal antiserum targeting amino acids 11–24 of Ank4_01 and of Ank4_02, which are identical paralogs, was generated by New England Peptide (Gardner, MA). HeLa cells were seeded in wells of a 6-well plate. The next day, when the cells were 90–95% confluent, 4 μg of pFlag-Ank4_01, pFlag-Ank9, or pFlag-BAP [bacterial alkaline phosphatase (Sigma-Aldrich)] DNA per well was transfected using Lipofectamine 2000 (Invitrogen) according to the manufacturer's directions. Between 18 and 24 h post transfection, 0.05% trypsin-EDTA (Invitrogen) was added to each well to promote cellular detachment and the cells were lysed in 300 μl of lysis buffer [20 μM Tris pH 7.4, 0.5 M NaCl, 0.7% Tween-20, with EDTA-free protease inhibitor (Roche Diagnostics GmBH, Mannheim, Germany)] for 40 min on ice. Insoluble proteins were pelleted at 10,000× g for 10 min at 4°C and discarded. Anti-Flag affinity gel beads (Sigma-Aldrich) were incubated with the cell lysates overnight at 4°C with rotation to precipitate Flag-tagged proteins. Resin was washed 4 times in lysis buffer prior to elution of proteins with 30 ul of 2X SDS sample buffer heated to 100°C. Samples were resolved by SDS-PAGE and transferred to nitrocellulose. The Western blots were screened with Ank4 antiserum (1:1000) or Flag antibody (1:1000; Sigma-Aldrich) followed by horseradish peroxidase (HRP)-conjugated goat anti-rabbit IgG (1:10,000, Cell Signaling Technology).

### Immunofluorescence detection of Ank4 expressed by *O. tsutsugamushi* in infected host cells

L929 cells were seeded onto glass coverslips in 24-well plates and treated with 0.4 μg/ml daunomycin (Sigma-Aldrich) followed by the addition of media laden with *O. tsutsugamushi* organisms that had been naturally released from infected L929 cells. Cells were fixed in 4% (vol/vol) PFA in PBS. Cells were methanol permeabilized for 30 s and blocked for 1 h at room temperature in PBS containing 5% (vol/vol) BSA. Next, the coverslips were incubated with fluorescein isothiocyanate-conjugated rat anti-*O. tsutsugamushi* serum (undiluted) and Ank4 antiserum (1:100) for 1 h at room temperature followed by incubation with Alexa Fluor 594-conjugated goat anti-rabbit IgG (1:1000; Invitrogen). Primary and secondary antibodies were diluted in PBS containing 5% and 1% (vol/vol) BSA, respectively. Coverslips were mounted using ProLong Gold antifade plus DAPI and imaged with a Zeiss LSM 700 laser-scanning confocal microscope.

## Results

### The *O. tsutsugamushi* genome encodes Anks that display characteristics of T1SS substrates

The *O. tsutsugamushi* Ikeda strain genome (NCBI accession number NC_010793.1) encodes 38 *ank* ORFs and 9 *ank* pseudogenes (Nakayama et al., 2008) that are distributed throughout the chromosome. SMART (Simple Modular Architecture Research Tool) analysis revealed that each carries up to 9 ankyrin repeats (Table 1). Eighteen of the 38 *ank* ORFs exist as multiple identical or near-identical paralogs and 12 occur as single copy genes. Ank18 carries no ankyrin repeats but is homologous to the non-ankyrin repeat portion of Boryong Ank1u7 (OTBS_1195), which does contain an ankyrin repeat domain (Cho et al., 2007), and was therefore included in this study. We selected a subset of the 38 full-length Anks that were substantially diverse in sequence such that they could be differentiated from each other by PCR. We chose all of the single copy Anks and one representative member of each multi-copy Ank group to arrive at a total of 20 Anks (Table 2). The selected Anks displayed characteristics of T1SS effectors, especially those encoded by other rickettsial pathogens (Table 3) (Delepelaire, 2004; Wakeel et al., 2011; Kaur et al., 2012; Thomas et al., 2014), but not T4SS substrates. The percentage of LDAVTSIF residues occurring within the 60 C-terminal amino acids of each Ank ranged from 37 to 63%. The pIs of 13 of the 20 full-length Anks were acidic, ranging between 4.6 and 6.3. Ank7_02, Ank10_01, and Ank15 had overall pIs of 7.3, 6.85, and 7.2, but had C-termini with pIs of 4.3, 5.0, and 4.2, respectively. All of the Anks had relatively few cysteines. Thus, *O. tsutsugamushi* Anks may be T1SS substrates.

**Table 1:** ***Orientia tsutsugamushi* Ikeda strain Anks**.

**Ank**	**Gene**	**Chromosomal coordinates^a^**	**Number of ankyrin repeats**	**Amino acid locations of ankyrin repeats per Ank protein**
Ank1_01	OTT_0036	(+) 44402–44929	0^b^	
Ank1_02	OTT_0753	(−) 780290–781279	3	23–52, 56–85, 89–118
Ank2	OTT_0049	(−) 54830–55864	4	1–30, 38–67, 71–100, 104–134
Ank3_01	OTT_0153	(−) 144257–144901	6	6–36, 40–69, 73–115, 119–149, 153–182, 186–213
Ank3_02^c^	OTT_0236	(−) 223718–224077	3	5–47, 51–81, 85–114
Ank3_03^c^	OTT_0237	(−) 224310–224588	2	12–42, 46–75
Ank3_04	OTT_0288	(−) 289650–290294	6	6–36, 40–69, 73–115, 119–149, 153–182, 186–213
Ank3_05	OTT_0316	(+) 329027-329575	5	8–37, 41–83, 87–117, 121–150, 154–181
Ank3_06	OTT_0982	(+) 1032433–1032981	5	8–37, 41–83, 87–117, 121–150, 154–181
Ank3_07^c^	OTT_1032	(−) 1072659–1073159	4	25–67, 71–101, 105–134, 138–165
Ank3_08	OTT_1112	(−) 1146364–1147071	5	6–36, 40–69, 73–115, 119–149, 153–182
Ank3_09	OTT_1118	(+) 1152471–1153019	5	8–37, 41–83, 87–117, 121–150, 154–181
Ank3_10^c^	OTT_1236	(−) 1271358–1271513	0	
Ank3_11	OTT_1453	(−) 1485654–1486298	6	6–36, 40–69, 73–115, 119–149, 153–182, 186–213
Ank3_12^c^	OTT_1465	(+) 1497782–1498282	4	25–67, 71–101, 105–134, 138–165
Ank3_13	OTT_1696	(−) 1721877–1722521	6	6–36, 40–69, 73–115, 119–149, 153–182, 186–213
Ank3_14^c^	OTT_1730	(−) 1751181–1751648	4	14–56, 60–90, 94–123, 127–154
Ank3_15	OTT_1852	(−) 1883248–1883895	6	7–37, 41–70, 74–116, 120–150, 1540183, 187–214
Ank4_01	OTT_0210	(−) 200588–201766	7	50–79, 83–112, 116–147, 151–180, 184–214, 218–279, 283–315
Ank4_02	OTT_1916	(+) 1951498–1952676	7	50–79, 83–112, 116–147, 151–180, 184–214, 218–279, 283–315
Ank5_01	OTT_0214	(+) 205067–206104	4	2–29, 36–65, 69–99, 103–132
Ank5_02	OTT_0412	(+) 432777–433814	4	2–29, 36–65, 69–99, 103–132
Ank5_03	OTT_0487	(−) 505323–506360	4	2–29, 36–65, 69–99, 103–132
Ank6_01	OTT_0226	(−) 215330–216340	4	23–52, 56–85, 89–118, 122–158
Ank6_02	OTT_1149	(+) 1180783–1181793	4	23–52, 56–85, 89–118, 122–158
Ank6_03	OTT_1496	(+) 1525158–1526168	4	23–52, 56–85, 89–118, 122–158
Ank6_04	OTT_1912	(−) 1947492–1948502	4	23–52, 56–85, 89–118, 122–158
Ank7_01^c^	OTT_0250	(−) 241933–242373	2	35–64, 100–130
Ank7_02	OTT_1509	(−) 1535130–1536386	3	35–64, 68–96, 100–129
Ank7_03	OTT_1936	(−) 1969541–1970797	3	35–64, 68–96, 100–129
Ank8	OTT_0257	(−) 252600–253769	5	1–31, 35–64, 68–98, 111–140, 144–173
Ank9	OTT_0298	(+) 307356–308624	7	21–51, 55–84, 88–117, 121–150, 154–188, 192–221, 225–256
Ank10_01	OTT_0398	(−) 416849–418504	9	20–50, 54–83, 87–117, 121–150, 154–184, 188–217, 222–252, 256–285, 290–319
Ank10_02	OTT_0875	(+) 918456–920111	9	20–50, 54–83, 87–117, 121–150, 154–184, 188–217, 222–252, 256–285, 290–319
Ank10_03^c^	OTT_1089	(−) 1122006–1122263	0	
Ank10_04^c^	OTT_1090	(−) 1122302–1122703	2	14–43, 48–78
Ank11	OTT_0459	(−) 482167–482853	2	10–39, 132–161
Ank12_01	OTT_0602	(−) 621330–622814	7	35–64, 68–97, 102–144, 148–178, 182–211, 215–246, 250–281
Ank12_02	OTT_1300	(−) 1318892–1320379	8	1–30, 35–64, 68–97, 102–144, 148–178, 182–211, 215–246, 250–281
Ank13	OTT_0852	(−) 899766–901238	8	18–48, 52–81, 85–114, 118–147, 151–182, 186–215, 219–254, 258–285
Ank14	OTT_1019	(+) 1063942–1065198	5	33–62, 66–95, 99–128, 132–161, 162–193
Ank15	OTT_1232	(+) 1268108–1269037	1	105–134
Ank16	OTT_1271	(+) 1296255–1297055	3	5–34, 41–93, 97–125
Ank17	OTT_1478	(+) 1505350–1506078	4	37–66, 69–98, 102–129, 133–163
Ank18^d^	OTT_1518	(−) 1548914–1549132	0	
Ank19	OTT_1519	(−) 1549361–1549852	4	4–33, 37–75, 79–110, 114–163
Ank20	OTT_1575	(+) 1603487–1605013	9	32–62, 66–96, 101–130, 135–164, 165–194, 200–233, 237–267, 271–300, 304–335

a *(+) and (−) refer to the sense and antisense DNA strands, respectively*.

b *Ank1_01 is homologous to the non-ankyrin repeat-containing portions of Ank1_02*.

c *These ORFs are annotated as probable pseudogenes by Nakayama et al. (2008)*.

d *Ank18 carries no ankyrin repeats, but exhibits homology to the non-ankyrin repeat portion of its homolog in the O. tsutsugamushi Boryong strain, Ank1u7 (OTBS_1195; Cho et al., 2007)*.

**Table 2:** ***O. tsutsugamushi* Anks selected for codon optimization and further analyses**.

**Ank**	**% Identity with its paralog(s)^a^**
Ank1_02	Ank1_01 (43.2%)
Ank2	Single copy
Ank3_08	Ank3_01 (83.4%), Ank3_04 (84.7%), Ank3_05 (69.8%), Ank3_06 (69.8%), Ank3_09 (69.8%), Ank3_11 (81.3%), Ank3_13 (84.7%), Ank3_15 (80.9%)
Ank4_01	Ank4_02 (100%)
Ank5_01	Ank5_02 (100%), Ank5_03 (100%)
Ank6_02	Ank6_01 (99.1%), Ank6_03 (100%), Ank6_04 (100%)
Ank7_02	Ank7_03 (100%)
Ank8	Single copy
Ank9	Single copy
Ank10_01	Ank10_02 (100%)
Ank11	Single copy
Ank12_01	12_02 (76.4%)
Ank13	Single copy
Ank14	Single copy
Ank15	Single copy
Ank16	Single copy
Ank17	Single copy
Ank18	Single copy
Ank19	Single copy
Ank20	Single copy

a *Pseudogenes were excluded*.

**Table 3:**
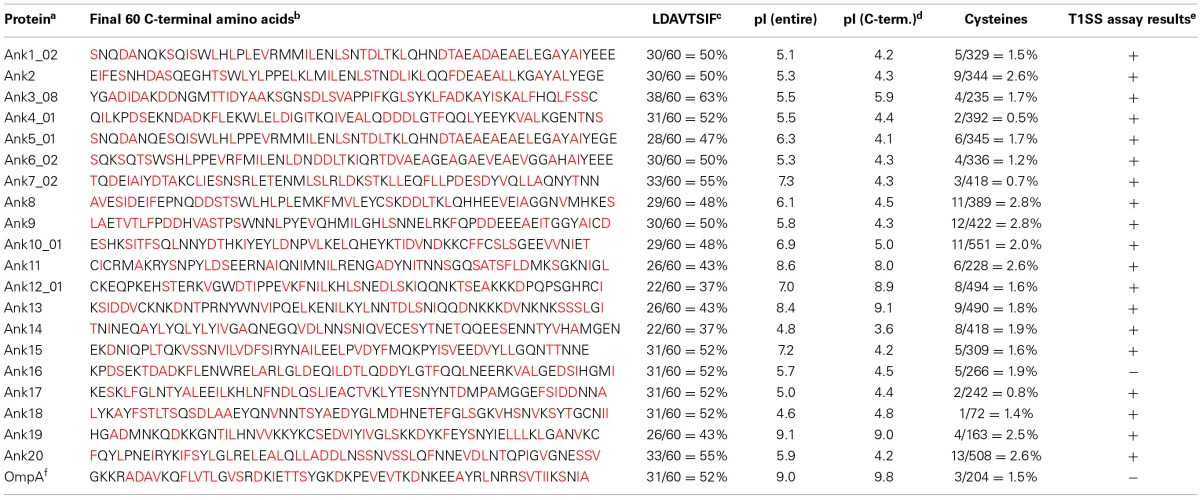
***O. tsutsugamushi* Ank C-termini features**.

*^a^Only the C-termini of Anks selected for codon optimization were analyzed*.

*^b^Red amino acids denote LDAVTSIF residues that are commonly found in the C-terminal secretion signals of T1SS substrates*.

*^c^The total number of LDAVTSIF residues per Ank was divided by 60 and multiplied by 100 to determine the percentage of LDAVTSIF residues in the final C-terminal 60 amino acids*.

*^d^C-term., C-terminus*.

*^e^(+) and (−) refer to whether or not E. coli secretes the chimeric HlyA-Ank protein in an HlyBD-dependent manner*.

*^f^OmpA C-terminal sequence was included because a chimeric HlyA-OmpA protein was a negative control for the T1SS secretion assay*.

### *O. tsutsugamushi* transcriptionally expresses Ank, T1SS, and T4SS genes during infection of mammalian host cells

The Ikeda and Boryong *O. tsutsugamushi* genomes encode VirB and VirD4 T4SS components (Cho et al., 2007; Nakayama et al., 2008). They also encode the T1SS components HlyB and HlyD which are sometimes annotated as AprD (alkaline protease secretion ATP-binding protein) and AprE (alkaline protease secretion protein), respectively, in other bacterial species (Lin et al., 2009; Kaur et al., 2012; Thomas et al., 2014) and are annotated as such in *O. tsutsugamushi* (Nakayama et al., 2008). *In silico* analyses of the *O. tsutsugamushi* T1SS components OTT_0076 (TolC), OTT_1107 (AprE), and OTT_1108 (AprD) revealed that they had 22, 27, and 41% amino acid identity to *E. coli* TolC, HlyD, and HlyB, respectively.

**Figure 1:**
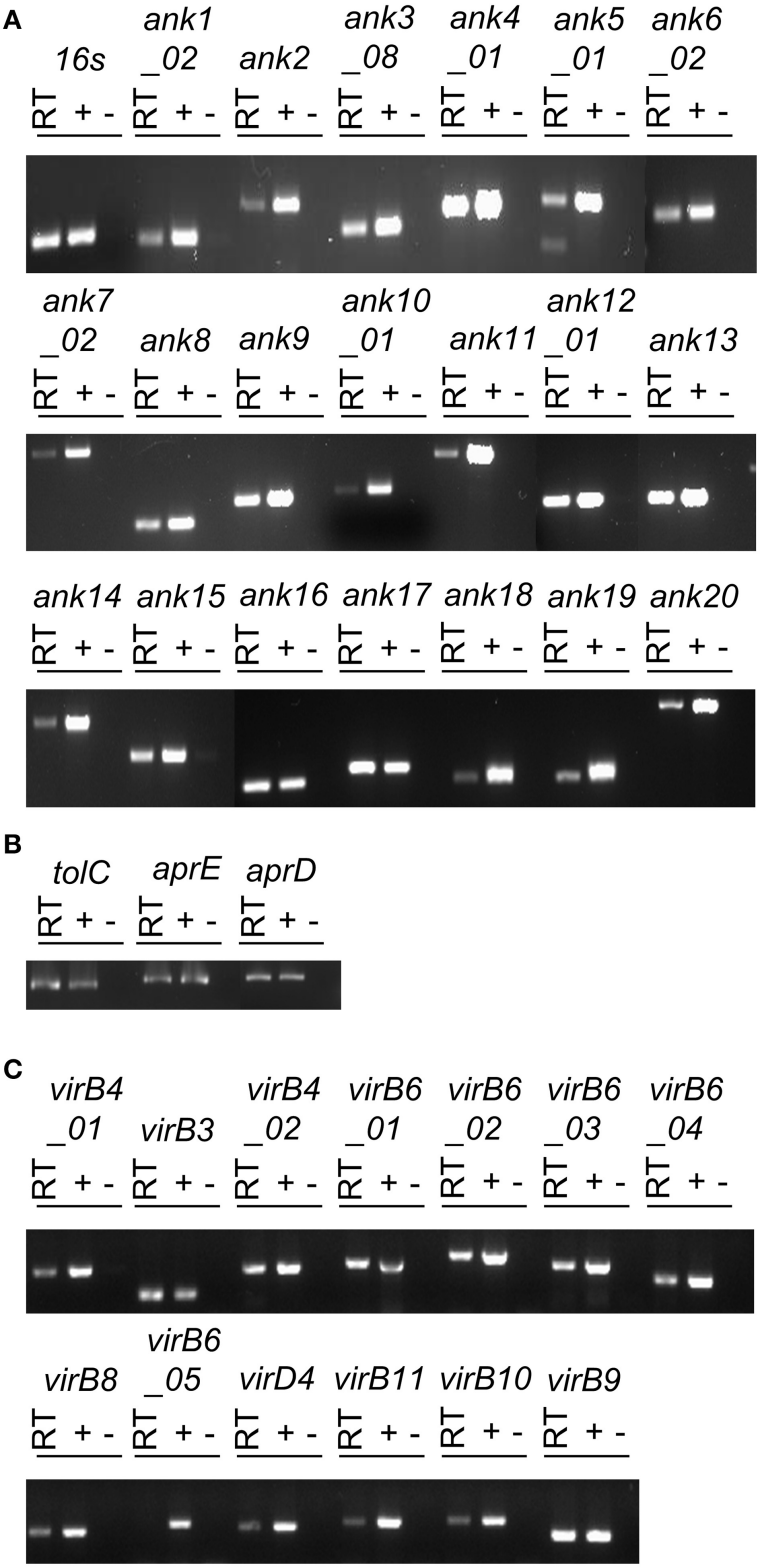
***O. tsutsugamushi* transcriptionally expresses Ank, T1SS, and T4SS genes during infection of mammalian host cells**. RT-PCR using primers targeting genes encoding *O. tsutsugamushi* Anks **(A)**, T1SS components **(B)**, T4SS components **(C)**, and the *16S rRNA* gene (infection control) **(A)** was performed on DNA-free total RNA isolated from infected L929 cells (RT lanes). *O. tsutsugamushi* genomic DNA and water served as positive (+) and negative (−) controls, respectively. Results are representative of two experiments with similar results.

To determine if *O. tsutsugamushi* transcribes T1SS and T4SS component genes and any of the 20 representative *ank* genes during infection, total RNA isolated from infected L929 cells was subjected to reverse transcriptase (RT)-PCR using gene-specific primers. Reactions performed in the absence of RT with primers specific for the *O. tsutsugamushi 16S rRNA* gene and host *ß-actin* yielded no PCR product (data not shown). Thus, the RNA templates were DNA-free. Reactions containing *O. tsutsugamushi* DNA or that lacked cDNA template were positive and negative controls, respectively. *O. tsutsugamushi 16S rRNA* primers were used to validate the amplification conditions. All *ank*, T1SS component, and T4SS component transcripts except for one of the five copies of *virB6* (OTT_1404) were detected in the reactions that contained RT (Figure 1). Sequencing was used to confirm the identities of representative amplicons (data not shown). Thus, *O. tsutsugamushi* expresses genes encoding Ank, T1SS, and T4SS components during infection of mammalian host cells.

### Assay for evaluating HlyBD-dependent secretion of potential T1SS substrates

We next sought to determine how *O. tsutsugamushi* Anks are potentially translocated. However, due to the pathogen's obligatory intracellular nature, no knock out-complementation system exists to directly assess the mechanism by which they are secreted. The Anks share features with T1SS substrates, including the presence of T1SS-like secretion signals in their C-termini. Accordingly, we evaluated *O. tsutsugamushi* Anks as putative T1SS effectors using the approach that previously verified *E. coli* strains K-12 or C600 could secrete recombinant forms of full-length *E. chaffeensis* or *R. typhi* T1SS candidate substrates in a TolC-dependent manner, respectively (Wakeel et al., 2011; Kaur et al., 2012). However, when full-length *O. tsutsugamushi* Anks were tested, they were toxic to *E. coli* C600, as the bacteria grew poorly or lysed regardless of inducer concentration, length of induction time, or induction temperature (data not shown). Also, for those *E. coli* C600 transformants that did not lyse, the Ank proteins were detected in the supernatants of both C600 and the C600 TolC-deficient mutant, which implied leaky secretion (data not shown). Thus, the TolC-dependent method using *E. coli* C600 was unsuitable for assessing *O. tsutsugamushi* Anks as potential T1SS substrates.

**Figure 2:**
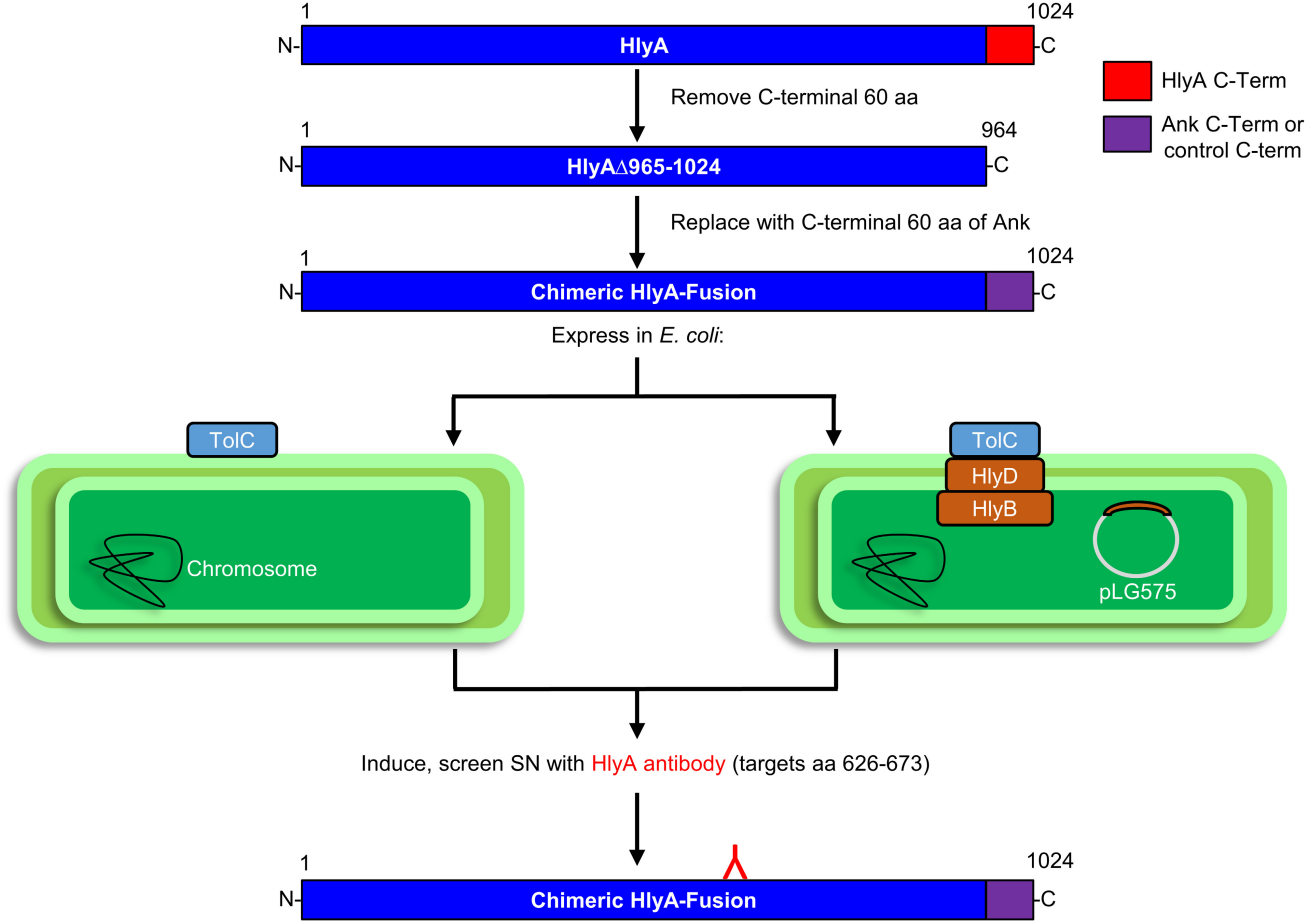
**Experimental approach for assaying *E. coli* secretion of HlyA chimeric proteins bearing the C-termini of putative T1SS substrates in an HlyBD-dependent manner**. *E. coli* BL21 (DE3) cells, which express TolC but not HlyB or HlyD, were transformed with pLG575 to constitutively express HlyB and HlyD and thereby functionally reconstitute the T1SS. *E. coli* with or without pLG575 were transformed with plasmids that express IPTG-inducible chimeric HlyA proteins having their 60 C-terminal residues (amino acids 964–1024) replaced with the 60–70 C-terminal residues of each *O. tsutsugamushi* Ank or control protein. The *E. coli* cultures were induced and centrifuged, and the resulting cell pellets and filter-sterilized supernatants were analyzed by Western blot using HlyA antibody to detect HlyA-fusion proteins that the bacteria expressed and secreted into the medium.

As an alternative, we developed an assay in which the C-terminal 60 amino acids of HlyA containing the T1SS secretion signal were replaced with the 60 C-terminal residues of *O. tsutsugamushi* Anks and tested for secretion in an HlyBD-dependent manner. This approach is outlined in Figure 2 and was based on a report that the C-terminus of *Mannheimia haemolytica* (formerly *Pasteurella hemolytica*) leukotoxin (LktA), a T1SS effector (Davies et al., 2002), would functionally replace the T1SS secretion signal of *E. coli* HlyA (Zhang et al., 1993, 1995). Moreover, our approach avoided the leaky TolC-independent secretion observed for *O. tsutsugamushi* Anks expressed in the C600/C600 TolC-deficient mutant system, as T1SS substrates are not directed to the TolC outer membrane channel in the absence of HlyB and HlyD (Thomas et al., 2014). *E. coli* strain BL21 (DE3) carries a chromosomal copy of *tolC*, but lacks *hlyB* and *hlyD*. This strain was complemented with plasmid pLG575 (Mackman et al., 1985) to constitutively express HlyB and HlyD and thereby reconstitute the T1SS.

**Figure 3:**
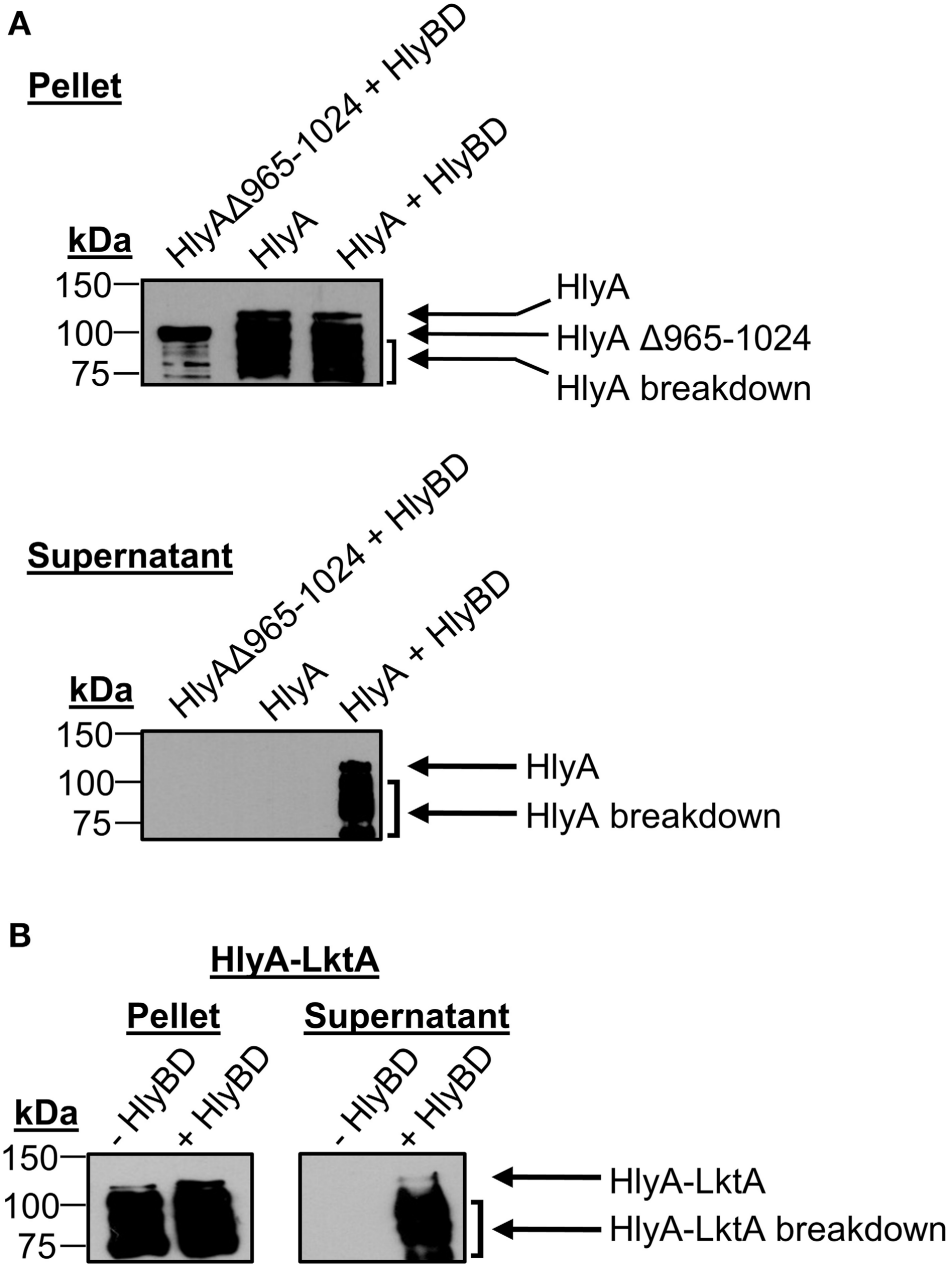
**Validation of the T1SS assay: *E. coli* secretion of HlyA and a chimeric HlyA-LktA protein depends on the substrates' C-terminal T1SS recognition signals**. *E. coli* BL21 (DE3) cells constitutively expressing HlyB and HlyD (+ HlyBD), or not (− HlyBD) were transformed with plasmids encoding full-length HlyA **(A)** HlyA lacking its 60 C-terminal amino acids (HlyAΔ965–1024) **(A)** or a chimeric HlyA-LktA protein corresponding to HlyAΔ965–1024 fused to the 70 C-terminal amino acids of *M. haemolytica* LktA **(B)**. Following IPTG induction of HlyA or HlyA chimeric protein expression, the cultures were centrifuged. The resulting *E. coli* cell pellets and filter-sterilized supernatants (SN) were analyzed by Western blot using HlyA antibody for the presence of HlyA proteins that had been expressed (Pellet) and secreted into the medium by the bacteria (SN), respectively. Arrows denote bands corresponding to the expected sizes for full-length and naturally occurring break down products of HlyA, HlyAΔ965–1024, and HlyA-LktA. Results are representative of at least three independent experiments.

To validate the system, we tested the ability of *E. coli* BL21 (DE3) to secrete HlyA and a chimeric HlyA protein with its C-terminal 60 amino acids replaced with the LktA C-terminal secretion signal (HlyA-LktA) (Zhang et al., 1993) in an HlyBD-dependent manner. *E. coli* expressing HlyA lacking the C-terminal secretion signal (HlyAΔ965–1024) served as a negative control. Following IPTG induction of HlyA protein expression, the supernatants were Western-blotted and screened with HlyA antibody for the presence of secreted HlyA proteins (Rowe et al., 1994). HlyA is a 107-kDa protein that after being produced quickly breaks down into a series of fragments that migrate with apparent molecular weights of ~70–100 kDa (Aldick et al., 2009). Bands corresponding to the expected sizes for full-length HlyA and its breakdown products, HlyAΔ965–1024, and HlyA-LktA were detected in the *E. coli* cell pellets (Figures 3A,B), thereby confirming their expression in an IPTG-inducible manner, as they were absent from non-induced samples (data not shown). Consistent with it lacking a T1SS secretion signal, HlyAΔ965-1024 was not detected in the supernatant from *E. coli* that also expressed HlyB and HlyD (Figure 3A). Full-length HlyA and chimeric HlyA-LktA bands were only detected in supernatants of *E. coli* that expressed HlyB and HlyD (Figures 3A,B), validating that *E. coli* BL21 (DE3) having a functionally reconstituted T1SS can be used as a tool to screen T1SS candidates.

### *E. coli* secretes chimeric HlyA proteins bearing the C-termini of *O. tsutsugamushi* Anks in an HlyBD-dependent manner

**Figure 4:**
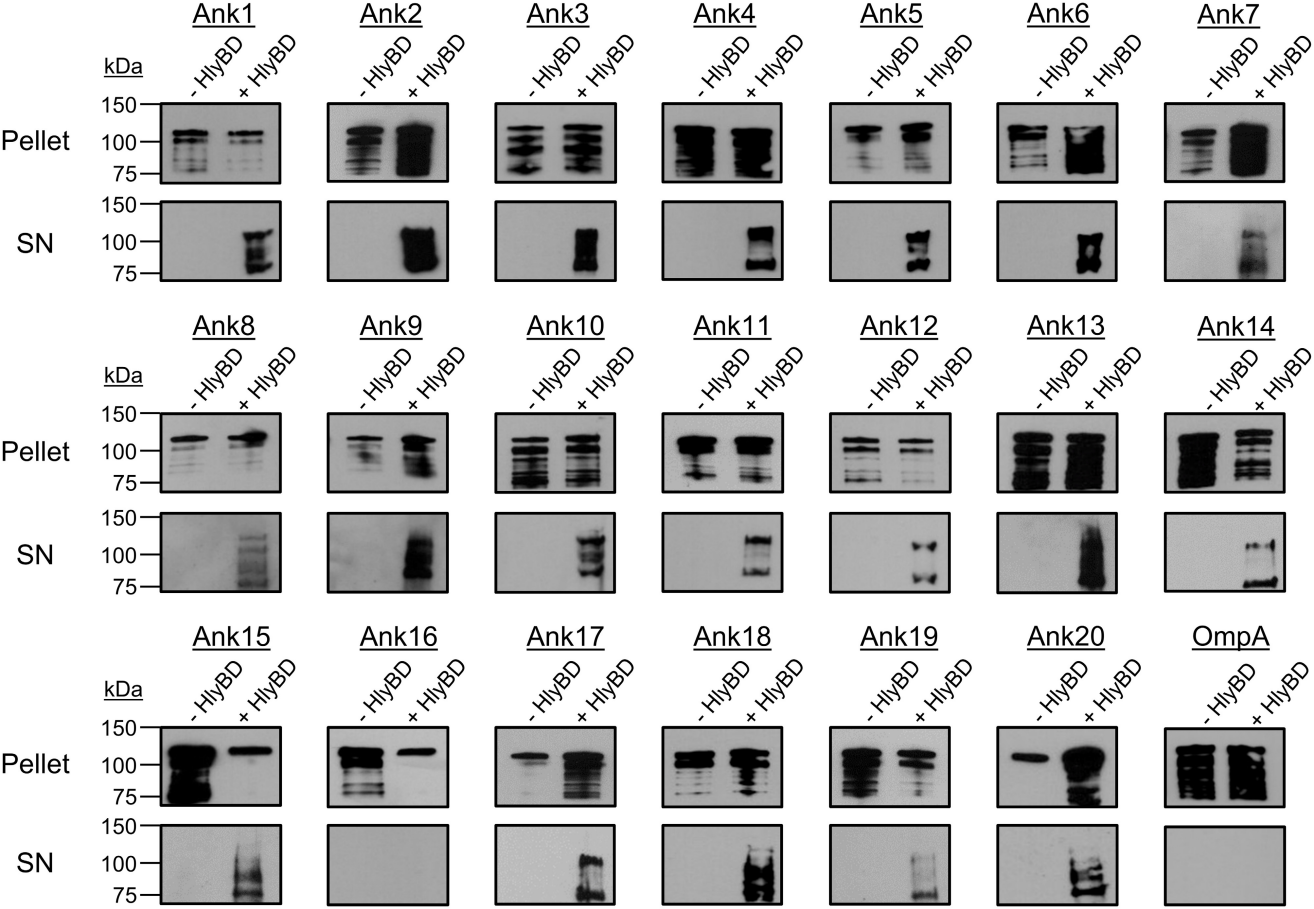
**Extracellular secretion of HlyA-Ank proteins from *E. coli* in an HlyBD-dependent manner**. *E. coli* BL21 (DE3) constitutively expressing HlyB and HlyD (+ HlyBD), or not (− HlyBD) were transformed with plasmids encoding the indicated chimeric HlyA-Ank protein or the negative control chimera, HlyA-OmpA. Following IPTG induction of HlyA chimeric protein expression, the cultures were centrifuged. The resulting *E. coli* cell pellets and filter-sterilized supernatants (SN) were analyzed by Western blot using HlyA antibody for the presence of HlyA chimeric proteins that had been expressed (pellet) and secreted into the medium by the bacteria (SN), respectively. Results per each HlyA-chimeric protein are representative of at least three independent experiments.

To evaluate the 20 Anks of interest as potential T1SS substrates, we assessed if swapping the final 60 amino acids of HlyA with the 60 C-terminal residues of each Ank would permit secretion of HlyA-Ank chimeras in an HlyBD-dependent manner. For these assays, HlyA and HlyAΔ965–1024 served as positive and negative controls, respectively. As an additional negative control to ensure that any secretion observed for HlyA-Ank proteins was specific to the inclusion of a T1SS-compatible sequence, we assayed a chimeric protein in which the HlyA C-terminal sequence was replaced with the 60 C-terminal amino acids of *O. tsutsugamushi* outer membrane protein A (HlyA-OmpA). The OmpA C-terminus is similar in LDAVTSIF composition (51.7%) to the C-termini of *O. tsutsugamushi* Anks and rickettsial effectors (Table 3) (Wakeel et al., 2011; Kaur et al., 2012), but is a cell envelope protein and, as such, should not be secreted. Following induction, all HlyA-Ank proteins and HlyA-OmpA were expressed, as HlyA antibody detected bands of the expected sizes for HlyA and/or its cleavage products in the *E. coli* cell pellets (Figure 4). With the exception of HlyA-Ank16 and HlyA-OmpA, all chimeric HlyA proteins were secreted in an HlyBD-dependent manner. The signals for secreted HlyA-Ank7_02, -Ank8, -Ank11, -Ank12_01, -Ank14, and -Ank19 were not as robust as for the other secreted HlyA-Anks. These data demonstrate that nearly all of the *O. tsutsugamushi* Anks bear C-terminal sequences that mediate heterologous recognition and secretion by the *E. coli* T1SS.

### The *C. burnetii* Dot/Icm T4SS cannot heterologously secrete *O. tsutsugamushi* Anks

**Figure 5:**
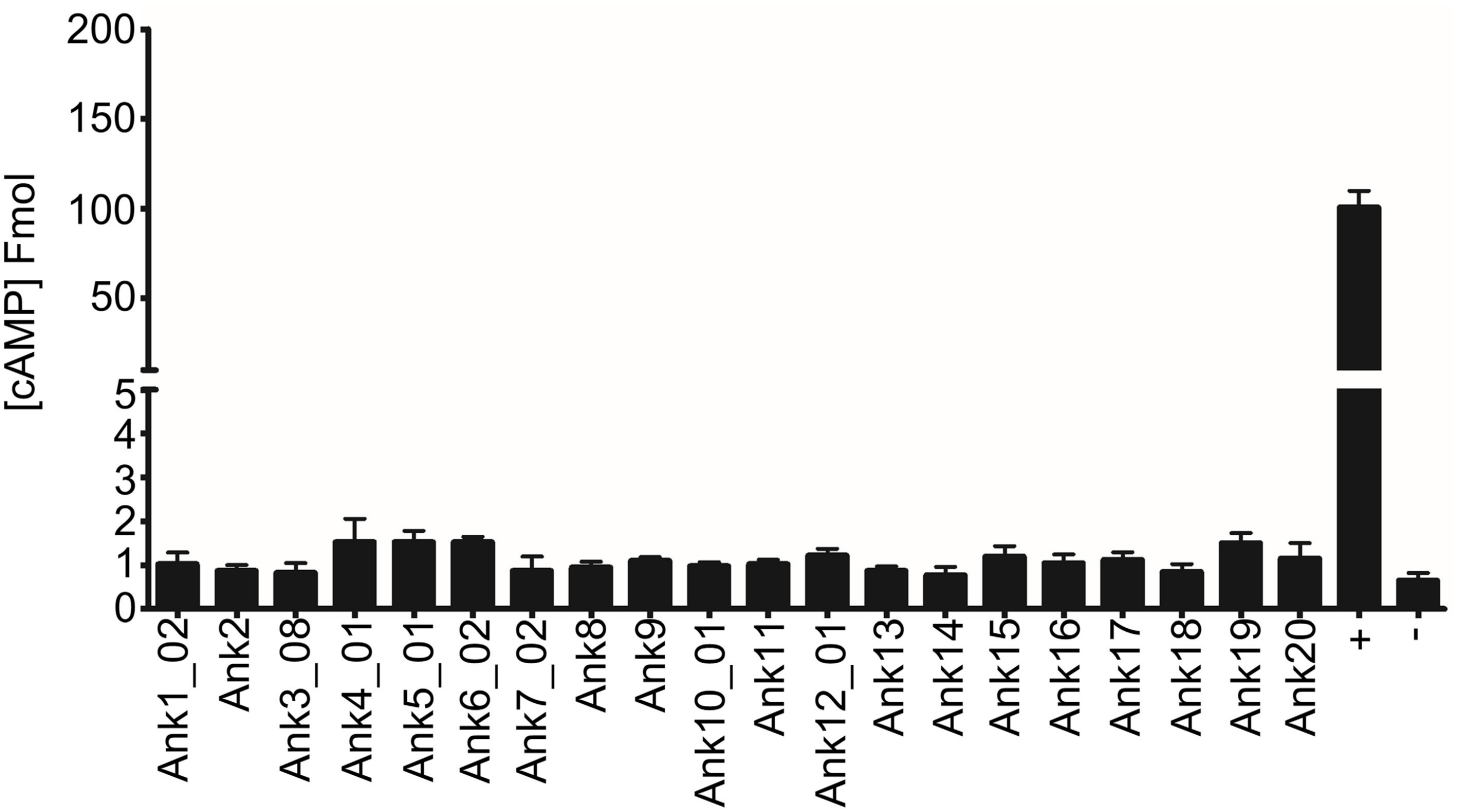
**The *C. burnetii* Dot/Icm T4SS cannot secrete *O. tsutsugamushi* Anks**. THP-1 macrophage-like cells were infected with *C. burnetii* transformants expressing CyaA-tagged *O. tsutsugamushi* Anks, CvpA (+), or CyaA alone (−). Bars represent the fold change in intracellular cAMP concentration for host cells infected with wild type *C. burnetii* transformants expressing CyaA-fusions relative to control cells infected with transformants expressing CyaA alone. Results are representative of two independent experiments.

Based on the HlyA-Ank secretion assay results and because of their C-terminal sequence characteristics, we rationalized that the *O. tsutsugamushi* Anks are not T4SS effectors. To validate this hypothesis, each of the 20 Anks of interest was N-terminally fused to the enzymatic reporter, *Bordetella pertussis* adenylate cyclase (CyaA) and expressed in the surrogate host, *C. burnetii*, which uses its Dot/Icm secretory apparatus to translocate T4SS substrates (Beare et al., 2011). Surrogate hosts that use the Dot/Icm system will secrete heterologously expressed VirB/VirD4 effectors, including the *Rickettsiales* effector, *A. phagocytophilum* AnkA (De Jong et al., 2008; Huang et al., 2010). THP-1 cells that had been differentiated into macrophage-like cells were infected with *C. burnetii* transformants expressing CyA-fusions of *O. tsutsugamushi* Anks, CyaA-CvpA (*Coxiella* vacuolar protein A; *bona fide* Dot/Icm effector; positive control) (Larson et al., 2013), or CyaA alone (negative control) and changes in host cAMP levels were quantified. Expression of each Cya-fusion protein in infected host cells was confirmed by Western blot analysis (data not shown). cAMP levels were considerably elevated relative to that in *C. burnetii* expressing CyaA alone when host cells were infected with *C. burnetii* expressing CyaA-CvpA, but not any CyaA-Ank (Figure 5). As expected, cAMP levels were low for host cells infected with transformants of a *C. burnetii* T4SS-defective DotA mutant. Thus, heterologously expressed *O. tsutsugamushi* Anks cannot be translocated by the *C. burnetii* Dot/Icm T4SS.

### Ectopically expressed *O. tsutsugamushi* Anks exhibit diverse subcellular localization patterns

**Figure 6:**
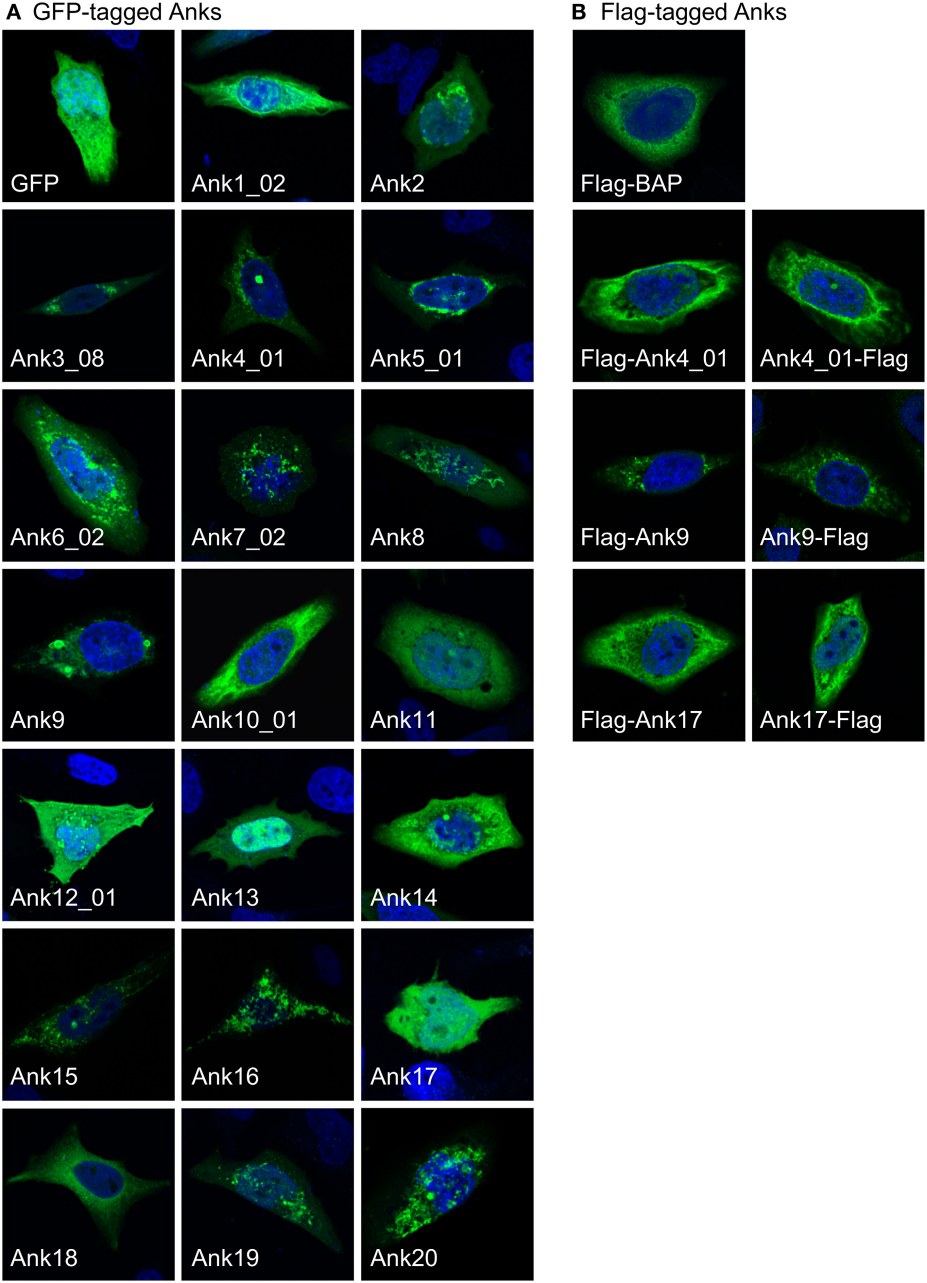
**Ectopically expressed *O. tsutsugamushi* Anks exhibit diverse subcellular localization patterns**. **(A)** HeLa cells expressing GFP alone or the indicated Ank proteins N-terminally fused to GFP were screened with GFP antibody, and visualized using confocal microscopy. **(B)** Subcellular localization patterns of ectopically expressed Anks are not affected by the fusion tag itself or tag placement. HeLa cells expressing the indicated Anks as N-terminally (Flag-Ank) or C-terminally Flag-tagged (Ank-Flag) fusion proteins or Flag-BAP were screened with Flag tag antibody and examined by confocal microscopy. **(A,B)** HeLa cell nuclei were stained with DAPI (blue). Representative images from 2 to 4 experiments performed per each ectopically expressed Ank are presented.

Upon secretion into host cells, bacterial effectors traffic to distinct subcellular locales where they exert their modulatory functions. As a first step in gaging the subcellular locations to where *O. tsutsugamushi* Anks potentially traffic, confocal microscopy was used to examine green fluorescent protein (GFP)-tagged Anks in HeLa cells. GFP-tagged Ank1_02, Ank2, Ank3_08, Ank4_01, Ank5_01, Ank6_02, Ank8, Ank10_01, Ank14, and Ank19 exhibited aggregative, perinuclear fluorescence patterns (Figure 6A). GFP-tagged Ank7_02, Ank9, Ank12_01, Ank15, Ank16, and Ank20 displayed vesicular and/or cytosolic aggregative staining patterns. GFP-Ank11 exhibited a reticulate distribution, while GFP-Ank13 localized predominantly in host cell nuclei but also was present in the cytosol. GFP-Ank17, GFP-Ank18, and GFP alone exhibited diffuse cytosolic patterns. To ensure that the GFP tag itself did not alter Ank subcellular trafficking, all Anks except for Ank10_01 and Ank14 were also expressed as N-terminal Flag-tag fusions and their fluorescence patterns in HeLa cells were assessed. Furthermore, to verify that fusion tag placement did not alter Ank subcellular trafficking, Ank4_01, Ank9, and Ank17 were expressed as C-terminal Flag-tagged forms. Flag-BAP served as an irrelevant bacterial protein control. N- and C-terminal Flag-tagged fusion Anks exhibited fluorescence patterns that were similar to those of their GFP-tagged counterparts (Figure 6B and data not shown). Thus, ectopically expressed *O. tsutsugamushi* Anks display diverse subcellular localization patterns, the most common of which was perinuclear/reticulate.

### Multiple *O. tsutsugamushi* Anks localize to the endoplasmic reticulum (ER)

**Figure 7:**
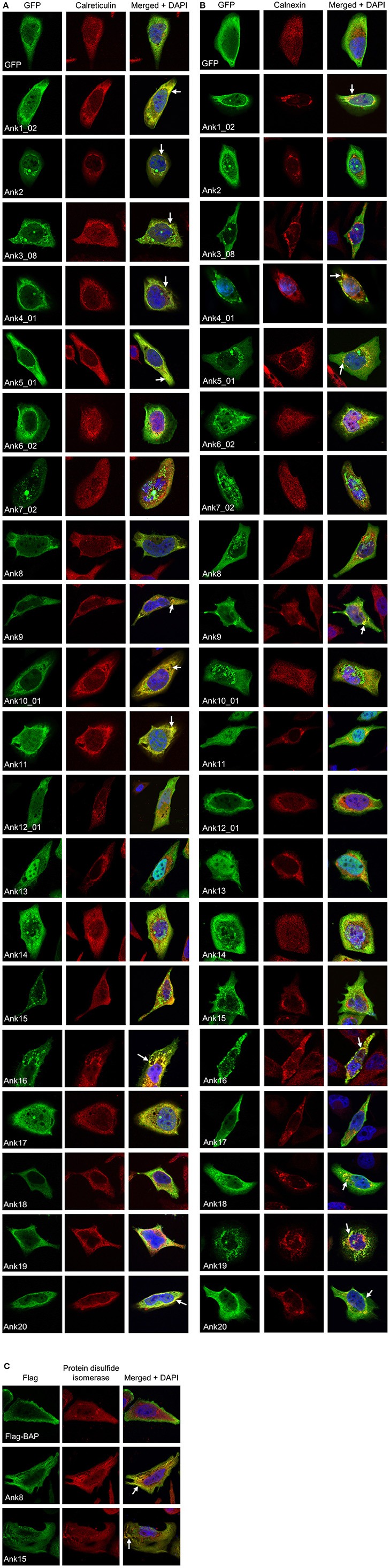
**Multiple ectopically expressed *O. tsutsugamushi* Anks localize to the endoplasmic reticulum**. HeLa cells expressing GFP alone or GFP-Anks were screened with GFP antibody, and cells expressing Flag-BAP or Flag-Anks were screened with Flag antibody. Additionally, cells were stained with antibody against either of the ER lumenal markers calreticulin **(A)** or protein disulfide isomerase **(C)**, or the ER transmembrane protein, calnexin **(B)** prior to examination by confocal microscopy. Representative fluorescence images of cells viewed for GFP (green), ER marker (red), and merged images plus DAPI (blue) are presented for each Ank from 2 to 4 independent experiments. Arrows denote representative areas of GFP and ER marker signal colocalization.

Given that multiple GFP-Anks yielded perinuclear or reticulate-like staining patterns reminiscent of the ER, we examined if the ectopically expressed Anks colocalized with ER markers. HeLa cells expressing GFP- or Flag-tagged Anks were screened with antibody against the ER lumenal protein, calreticulin, or the ER transmembrane protein, calnexin. GFP-tagged Ank1_02, Ank4_01, Ank5_01, Ank9, Ank16, and Ank20 exhibited varied degrees of colocalization with both calnexin and calreticulin (Figures 7A,B; Table A8). GFP-tagged Ank2, Ank3_08, Ank10_01, and Ank11 colocalized exclusively with calreticulin, while GFP-Ank18 and GFP-Ank19 colocalized only with calnexin. Neither GFP-tagged nor Flag-tagged versions of Ank8 and Ank15 colocalized with calreticulin or calnexin. However, Flag-Ank8 and Flag-Ank15 colocalized with the ER lumenal marker, protein disulfide isomerase (Figure 7C). GFP-tagged ER-tropic effectors Ank1_02, Ank2, Ank3_08, Ank4_01, Ank16, and Ank19 pronouncedly affected ER morphology, as the organelle in HeLa cells overexpressing these Anks was distended, collapsed, and/or fragmented into aggregated vesicles that were positive for calnexin/calreticulin and GFP. GFP-tagged ER-tropic effectors Ank9, Ank10_01, and Ank20 affected ER morphology to a lesser extent, as the ER staining patterns in these cells was largely of the characteristic reticulate morphology consistent with an intact ER, with some GFP- and ER marker-dual positive vesicular fragments present. GFP-tagged Ank6_02, Ank7_02, Ank12_01, Ank13, Ank14, and GFP alone neither colocalized with ER markers nor altered ER morphology (Figures 7A,B). Taken together, these data indicate that multiple ectopically expressed *O. tsutsugamushi* Anks localize to the ER and/or alter its morphology.

### *O. tsutsugamushi* expresses Ank4 during infection of mammalian host cells

**Figure 8:**
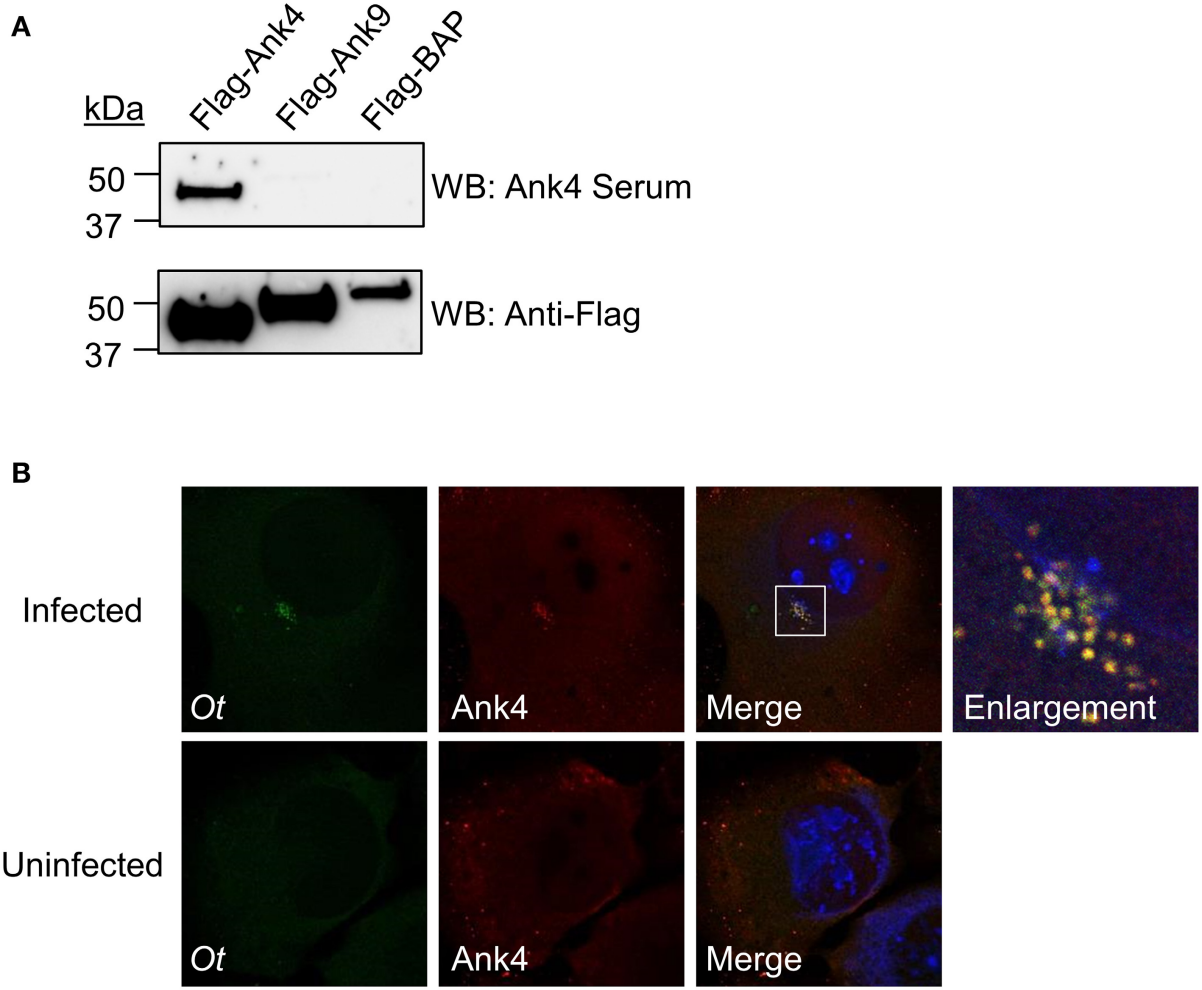
***O. tsutsugamushi* expresses Ank4 during infection of mammalian host cells**. **(A)** Ank4 antiserum specifically recognizes its target. Western blots of Flag-tagged Ank4, Ank9, or BAP that had been immunoprecipitated from transfected HeLa cells were screened with Ank4 antiserum and Flag tag antibody. Data presented are representative of three experiments with similar results. **(B)** Ank4 immunofluorescent signal colocalizes with *O. tsutsugamushi*. L929 cells infected with *O. tsutsugamushi* were screened with antisera specific for the bacterium (*Ot*; green) and Ank4 (red) were examined by laser-scanning confocal microscopy. Host cell nuclei were stained with DAPI (blue). Data presented in **(A)** and **(B)** are each representative of three experiments with similar results.

Because all experiments performed thus far involved heterologous or ectopically expressed Anks, we evaluated if *O. tsutsugamushi* expressed a representative Ank during infection of mammalian host cells. We generated antiserum specific for Ank4_01 and Ank4_02 (identical paralogs; Table 2) residues 11–24. To verify the specificity of the Ank4 antiserum, Flag-tagged proteins were immunoprecipitated from whole cell lysates of HeLa cells expressing Flag-Ank4, -Ank9, or -BAP and Western blotted with Ank4 antiserum or Flag antibody. Flag antibody detected bands of the expected size for each Flag-tagged protein, thereby demonstrating that each had been expressed and precipitated (Figure 8A). Ank4 antiserum was specific for its target, as it detected Flag-Ank4 but neither Flag-Ank9 nor Flag-BAP. Confocal microscopic analysis of infected L929 cells using Ank4 antiserum in conjunction with anti-*O. tsutsugamushi* serum detected Ank4 signal specifically colocalized with *O. tsutsugamushi* organisms (Figure 8B). If secreted Ank4 was present in the infected host cells, it was at a concentration below the level of detection. These data confirm that *O. tsutsugamushi* translationally expresses Ank4 during infection of mammalian host cells.

## Discussion

As an intracellular pathogen, *O. tsutsugamushi* has evolved to modulate eukaryotic host cell functions to facilitate its intracellular survival. Yet, the virulence factors that enable it to accomplish this feat are poorly defined or understood. In this study, we inferred the importance of the T1SS and family of Ank effectors to *O. tsutsugamushi* pathogenesis. The *O. tsutsugamushi* Ikeda strain expressed all T1SS component genes and all 20 distinguishable *ank* genes during infection of L929 cells, which implies the relevance of the encoded products. A recent transcriptional analysis of 9 of the 50 *ank* genes encoded by the *O. tsutsugamushi* Boryong strain revealed that all nine are transcribed during infection of mammalian host cells (Min et al., 2014). Thus, both *O. tsutsugamushi* strains express multiple *ank* genes during infection. Also, pooled scrub typhus patient sera was shown to weakly recognize recombinant forms of three of the nine Boryong Anks, indicating that some Anks are potentially expressed during human infection and may elicit a weak humoral immune response (Min et al., 2014). *O. tsutsugamushi* Ikeda expressed Ank4 during infection of L929 cells. Because Ank4 was not detected elsewhere in infected host cells, we cannot conclude whether the bacterium translocates Ank4. However, our inability to detect secreted Ank4 is not surprising, as bacterial effectors do not need to be delivered into host cells at high abundances to perform their functions. Moreover, several lines of evidence argue for the likelihood that *O. tsutsugamushi* translocates Anks into host cells: (1) the *O. tsutsugamushi* Anks strongly resemble T1SS effectors; (2) the *O. tsutsugamushi* genome has retained genes encoding one of the largest Ank repertoires of any organism and the T1SS over the course of its reductive evolution (Nakayama et al., 2008; Jernigan and Bordenstein, 2014); and (3) Anks that have been examined for translocation by genetically tractable organisms using knock out-complementation approaches have proven to be *bona fide* effectors (Al-Khodor et al., 2008; Pan et al., 2008; Habyarimana et al., 2010; Luhrmann et al., 2010).

The T1SS translocates substrates directly into the extracellular milieu or, in the case of an intracellular bacterium such as *O. tsutsugamushi*, directly into the host cell cytosol where the secreted effectors would have direct access to their host cell targets. T1SS secretion signals are not highly conserved, but known T1SS effectors share trends in their primary sequences (Thomas et al., 2014). *In silico* analyses of the Ikeda Anks identified them as potential T1SS effectors, as 37–63% of the residues in their final 60 amino acids consisted of LDAVTSIF residues, most had overall acidic pIs or had acidic C-termini, and most had very few cysteines. These sequence characteristics are similar to those of *E. chaffeensis* tandem repeat protein (TRP) 32, TRP47, TRP120, and Ank200, and *R. typhi* RARP-1 (*Rickettsia* ankyrin repeat protein 1), the latter two of which are Anks. These rickettsial proteins were proven to be T1SS substrates using *E. coli* C600 or K-12 as a heterologous host to secrete them in a TolC-dependent manner (Wakeel et al., 2011; Kaur et al., 2012). However, *O. tsutsugamushi* Anks were toxic to *E. coli* C600 and thus were incompatible. To circumvent this issue, we evaluated the ability of *E. coli* BL21 (DE3) to secrete chimeric HlyA-Ank proteins in an HlyBD-dependent manner, which (1) eliminated the toxicity associated with full-length Anks; (2) avoided leaky secretion because T1SS substrates cannot be translocated from the cytosol in the absence of HlyB and HlyD (Thomas et al., 2014); and (3) enabled us to directly assess the compatibility of each Ank protein's C-terminal 60 amino acids to recognition by heterologous T1SS machinery. This approach identified 19 of the 20 distinguishable Anks as possible T1SS substrates. Only HlyA-Ank16 was not secreted. Ank16 displays characteristics that are comparable with the other T1SS-positive Anks: 52% of its C-terminal 60 residues are LDAVTSIF amino acids, it has a pI of 5.7, and only 1.9% of its residues are cysteines. Yet, these primary sequence characteristics alone are not enough for Ank16 to be recognized and translocated by a functionally reconstituted T1SS. Consistent with this observation, even though the final 60 amino acids of OmpA share primary sequence characteristics with *O. tsutsugamushi* Anks and other T1SS effectors, *E. coli* cannot secrete HlyA-OmpA in an HlyBD-dependent manner.

Heterologously expressed Anks could not be secreted by *C. burnetii* in a Dot/Icm-dependent manner. While it cannot be absolutely ruled out that this result is simply due to incompatibility of the Dot/Icm apparatus with VirB/VirD4 translocation signals, we view this possibility as being highly unlikely for three reasons. First, *O. tsutsugamushi* Ank C-termini more closely resemble translocation signals of T1SS vs. T4SS substrates. Second, all but one of the Ank sequences tested were compatible with a T1SS apparatus and, to our knowledge, bacterial effectors are specific for a particular secretion system. Third, Dot/Icm systems have already been proven to secrete VirB/VirD4 substrates (De Jong et al., 2008; Huang et al., 2010).

Anks of intracellular bacterial pathogens have been shown to mediate key interactions with host cell factors that aid bacterial survival. *L. pneumophila* AnkX prevents microtubule-dependent vesicular transport, blocking fusion of the *Legionella*-containing vacuole (LCV) with late endosomes (Pan et al., 2008). AnkX also posttranslationally modifies Rab1 and Rab35, which is critical for proper LCV biogenesis (Mukherjee et al., 2011). *L. pneumophila* AnkB co-opts host polyubiquitination machinery to facilitate bacterial survival in macrophages and amoeba, and is necessary for intrapulmonary dissemination in mice (Price et al., 2009), while AnkH and AnkJ are essential for pathogen proliferation in host cells (Habyarimana et al., 2010). *C. burnetii* AnkG interacts with mammalian host protein p32 to delay pathogen-induced apoptosis (Luhrmann et al., 2010). Other *C. burnetii* Anks localize to microtubules, mitochondria, and the parasitophorous vacuole membrane (Voth et al., 2009). *A. phagocytophilum* AnkA interacts with host cell tyrosine kinases Abl-1 and Src, which, in turn, enables its interaction with host SHP-1, an interaction that contributes to pathogen survival (Ijdo et al., 2007; Lin et al., 2007). AnkA also translocates to the host cell nucleus where it binds the promoter of the antimicrobial gene, *CYYB* to inhibit host cell oxidative killing (Garcia-Garcia et al., 2009). Lastly, *E. chaffeensis* Ank200 also traverses to the host cell nucleus where it may globally alter host cell gene expression (Zhu et al., 2009).

To assess where *O. tsutsugamushi* Anks potentially traffic during infection, we examined the subcellular localization patterns of ectopically expressed Anks in HeLa cells. Of the 20 Anks evaluated, 14 trafficked to the ER and some of these promoted fragmentation, distension, and/or collapse of the organelle; one localized to the nucleus; and the other five either remained in the cytosol as diffuse or vesicular aggregates of unknown origin. Thus, *O. tsutsugamushi* Anks traffic to distinct subcellular locales where they likely modulate host cell processes to the pathogen's advantage. The nuclear localization of Ank13 suggests that it, like *A. phagocytophilum* AnkA, may globally regulate host cell transcription. While many of the ER-tropic Anks accumulated in both the ER lumen and at the ER membrane, Ank3_08, Ank8, Ank10_01, Ank15, and Ank20 specifically localized in the lumen and Ank18 and Ank19 trafficked directly to the ER membrane. Thus, subsets of ER-tropic Anks may traffic to distinct locales within the ER. The mechanism responsible for these Anks' ER-tropism is unknown, as sequence analysis failed to find a KDEL ER-retention signal in any ER-tropic Ank (data not shown). Nevertheless, it can be inferred that this tropism is specific and not an artifact of overexpression because several other ectopically expressed Anks trafficked to non-ER sites.

The subcellular trafficking patterns observed for all recombinant Anks were specific to the Ank portions and not the fusion tag, as differentially tagged Anks exhibited the same localization patterns irrespective of fusion tag placement or the tag itself. Results for the Ikeda Anks obtained here largely contrast those reported for the nine ectopically expressed Boryong Anks, five of which accumulated exclusively in host cell nuclei, three of which localized to both the cytosol and nuclei, and one of which remained in the cytosol. It was not examined if the Boryong cytosolic Anks colocalized with ER markers, but staining for early endosomal and lysosomal markers revealed no colocalization with Anks (Min et al., 2014).

The ER is the site of protein, carbohydrate, and lipid synthesis, as well as the site of assembly of molecular complexes involved in antigen presentation (Roy, 2002). What advantage could *O. tsutsugamushi* gain by directing such a large portion of its Ank repertoire to the ER and, conversely, how might this phenomenon affect the host response to infection? Possible clues to answer these questions arise when one considers that multiple bacterial pathogens target the ER. *L. pneuomphila, Brucella* spp., and *Chlamydia* spp. replicate in parasitophorous vacuoles that intercept ER-derived traffic, fusing with, and acquiring characteristics of ER membranes (Kagan and Roy, 2002; Celli et al., 2003; Robinson and Roy, 2006; Arasaki et al., 2012; Dumoux et al., 2012; Hubber et al., 2014). Several *Brucella* spp. effectors traffic to the ER, causing its structural reorganization, inducing ER stress and the unfolded protein response (UPR), and inhibition of protein secretion (Myeni et al., 2013; Smith et al., 2013). *Streptococcus pyogenes* injects toxins into host cells to generate ER stress and the UPR, which, in turn, leads to overproduction of asparagine. The released asparagine activates a *S. pyogenes* transcriptional profile that promotes bacterial growth and colonization (Baruch et al., 2014). The *Brucella melitensis* effector, TcpB induces the UPR to promote bacterial survival (Smith et al., 2013). Yet, the *B. abortus* ER-tropic effector, VceC, which also induces the UPR, is required for *B. abortus-induced* inflammatory cytokine production (Myeni et al., 2013). Likewise, the ER stress signaling pathway induced in response to *Mycobacterium tuberculosis* has been implicated in controlling the pathogen's growth (Lim et al., 2011). Thus, *O. tsutsugamushi* ER-tropic Anks conceivably mediate host-pathogen interactions that favor parasitism and/or pathogen detection and the ensuing antimicrobial response.

In closing, this study adds to the growing appreciation for the T1SS as an important translocation system for rickettsial pathogens and reveals the potential that the *O. tsutsugamushi* Ank armamentarium has for enabling the bacterium to parasitize mammalian host cells. Moving forward, it will be important to determine if the bacterium expresses Ank proteins in addition to Ank4 during infection and to elucidate the functional benefits that these virulence factors afford this understudied pathogen.

## Disclaimer

The views expressed in this article are those of the authors and do not necessarily reflect the official policy or position of the Department of the Navy, Department of the Defense, or the U.S. Government. As an employee of the U.S. Government (Allen L. Richards) this work was prepared as part of his official duties and therefore because of Title 17 U.S.C paragraph 105 provides that “Copyright protection is not available for any work of the U.S. Government,” this article cannot be copyrighted.

### Conflict of interest statement

The authors declare that the research was conducted in the absence of any commercial or financial relationships that could be construed as a potential conflict of interest.
