# Supplementary material for: Orientia tsutsugamushi ankyrin repeat-containing protein family members are Type 1 secretion system substrates that traffic to the host cell endoplasmic reticulum
Source: Front Cell Infect Microbiol. 2015 Feb 3;4:186. doi: 10.3389/fcimb.2014.00186 (PMC4315096; doi:10.3389/fcimb.2014.00186)
Supplement: Supplementary file 1 [file DataSheet1.DOCX]

***Supplementary Material***

***Orientia tsutsugamushi* Ankyrin Repeat-Containing Protein Family Members are Type 1 Secretion System Substrates that Traffic to the Host Cell Endoplasmic Reticulum**

**Lauren VieBrock^1,4^, Sean M. Evans^1,4^, Andrea R. Beyer^1^, Charles L. Larson^3^, Paul A. Beare^3^, Hong Ge^2^, Smita Singh^1^, Kyle Rodino^1^, Robert A. Heinzen^3^, Allen L. Richards^2^, and Jason A. Carlyon^1^***

^1^Department of Microbiology and Immunology, Virginia Commonwealth University School of Medicine, Richmond, Virginia, USA

^2^Viral and Rickettsial Diseases Department, Naval Medical Research Center, Silver Spring, Maryland, USA

^3^Coxiella Pathogenesis Section, Rocky Mountain Laboratories, National Institute of Allergy and Infectious Diseases, National Institutes of Health, Hamilton, Montana, USA

^4^L.V. and S.M.E. contributed equally to this work.

*** Correspondence:** Department of Microbiology and Immunology, Virginia Commonwealth University School of Medicine, P.O. Box 980678, Richmond, Virginia, 23298-0678, USA. jacarlyon@vcu.edu.

1. **Supplementary Data**
2. **Supplementary Figures and Tables**

## Supplementary Tables

Table A1. Oligonucleotides used for RT-PCR

| Designation^1^ | Sequence (5’ to 3’) |
| --- | --- |
| Ot *16S*-911F | GTGGAGCATGCGGTTTAATTCGATGATC |
| Ot *16S*-1096R | TAAGAATAAGGGTTGCGCTCGTTGC |
| *tolC*-581F | CAAGTGTTAGTGTAGCTAATGCACAAGTTC |
| *tolC*-908R | GGCTGAAAAGGTAAAGGGCTAATTACTG |
| *aprE*-914F | ACAGTACTCAGCAAGATATCGAACGAAC |
| *aprE*-1293R | CGATACTAGCACTACCCTCCCAGTAAAC |
| *aprD*-985F | AAGGCTATGCCTATGCTAAAGGTAACTG |
| *aprD*-1293R | ATTCAACACCCTGAGGAAGATAACCTAC |
| *virB4_01*-1687F | AGTGCTGGAGCTAGTGTAAAGAAGC |
| *virB4_01*-1964R | CTGGAGAGGCTAGTAAGCTGAATTT |
| *virB3*-36F | AGGGTTAACTAGGCCTCCAATGAT |
| *virB3*-182R | GCAAATACCCTACCCCATGTACTACTAATG |
| *virB4_02*-1956F | AGATGAGGCATGGGCTTTGATTG |
| *virB4_02*-2228R | ACCGTGAACCAGGATCTGTGG |
| *virB6_01*-527F | GGGATGCTGTTGCAAGAGCATGGC |
| *virB6_01*-834R | CCATTTCCGGTATGGCATACTGGCTTTG |
| *virB6_02*-90F | GTTGCTAGTTATTGTACTGCCATCCATTGC |
| *virB6_02*-451R | CGCAGCCCACCTAGCATTACCTTC |
| *virB6_03*-626F | ACGGAGGGGTTGAGAATACCGAAATAGG |
| *virB6_03*-929R | CTGGGCATACCGGCTTTGATCCTGA |
| *virB6_04*-2220F | ATTGATGTTAGTTGCAGTGGAAGC |
| *virB6_04*-2441R | CTCCTGCTAACGAACCTGCA |
| *virB8*-92F | ACCCATTGCTACATCGGTCTGTAG |
| *virB8*-360R | TGACTAGCAAGTTGACTGTAGTTGTAAC |
| *virB6_05*-1205F | GCATATTAGGGCTGATGATTGCTCC |
| *virB6_05*-1517R | CGACAAACGCAATGCCAGCAAATAATA |
| *virD4*-837F | TGGTGAAGTGGTTCGTACTATGCG |
| *virD4*-1773R | CAGGTGTTAAGCCAACGTATATTGTTG |
| *virB1*-77F | TGATGATTAACCGCCCTGGTGAG |
| *virB1*-990R | CAATCGCCGTGTAATCAAATGCTC |
| *virB10*-870F | TTGGTCAGAAGTACAGATTGCAGGTAG |
| *virB10*-1530R | CGACTCATACCGCAAATCTCTGTT |
| *virB9*-153F | GCATGCTGGATTTCAATCAAGTATTGAG |
| *virB9*-438R | GTTCACGCCCTACTTCTAGCTCC |
| *ank1_02*-21F | TCGCGCTCACCTACTATATGTTACTACAAGAAG |
| *ank1_02*-193R | CAGCAATATGTAATGCAGTGCTTCCACG |
| *ank2*-433F | GAATGTCACACGATGTTTATGGCAGATCC |
| *ank2*-682R | TATCCCAAACAGATAAGCCACTTTCGTTAACG |
| *ank3_05*-123F | TGGGCACACTGCTTTACATCTTGTTG |
| *ank3_05*-309R | AGGTTCTATACATCTGCATTCAACAGCATCAG |
| *ank4*-122F | CATGAGGGTGGCTTTAGTCTTGCTG |
| *ank4*-349R | CGTCTGTACATGGTACATTGACATTAGCTCC |
| *ank5*-204F | ATATGGCCATACTGCTTCTCATTACGCTG |
| *ank5*-442R | ACATCCTGTAAGCGTCTGCAAATGG |
| *ank6*-21F | TCGTGCTCGCCTATTATATGCTACTGC |
| *ank6*-192R | AGCAACATGTAACGCAGTGTTGTCC |
| *ank7*-303F | GCAAACTATTCTGCATATTGCAGCTCAGG |
| *ank7*-612R | AGGCCAGTTTAAAGCCATACCAGC |
| *ank8*-113F | CTCCTTTATATTGCGCAGCAAAGGAAGG |
| *ank8*-295R | CTACATTAGTTGTTTGAGCTGCGTGAGC |
| *ank9*-379F | CATTATGCTGCTCGACATGGTTTGCC |
| *ank9*-613R | TGTGACAGATAACAGCACGATGTAAAGGAG |
| *ank10*-1319F | CTGAAGTCGCAAGAACAATTGAGAATGGAAC |
| *ank10*-1573R | CATGCTGAAGCTCTTTCAAAACAGGGTTG |
| *ank11*-141F | CATATGCATGCTTGCACAGCGTAAGTC |
| *ank11*-543R | CGAATAACGTTTTGCCATCCTGCATATGC |
| *ank12_01*-335F | CCAGACCGTACACTCACCAAGG |
| *ank12_01*-570R | AGCACGATGCAAAGCAGTATCACC |
| *ank13*-1051F | CGCGCAGCACAACATAAACAGG |
| *ank13*-1288R | GTGCGCTTTGCATCAGCTTTCG |
| *ank14*-463F | GCAGGTGCAGATCCTGCTCTAAC |
| *ank14*-801R | CTTCTCCTGATCAAAGTGATATGGAAGATCCTG |
| *ank15*-94F | ACTCCAGAAATTGCTACTGATTTGCTACCC |
| *ank15*-344R | CTAGCTGCAGTATGTAGGATAGTTCTGCC |
| *ank16*-460F | GATAAGCATGATGATGGGATTGCAGAGC |
| *ank16*-703R | ATATCTGCTCATCCAGTCCGAGACG |
| *ank17*-202F | GCAGGTGGAATACATGCTTTACGTTGG |
| *ank17*-450R | GTCAGCATCTATGTGATTACTCTCCACAGC |
| *ank18*-2F | TGGATGATGCTACCAGTAGCAAAATTATCGATATTC |
| *ank18*-219R | TTAAATAATATTGCAGCCAGTGTAGCTTTTTACATTGC |
| *ank19*-107F | ATGATGGCCAAACTCCTTTGCATAGAG |
| *ank19*-273R | CCAGCAATCTATGGCATGACTGAATGG |
| *ank20*-131F | GCAATGTTGCAGCTGTTGAACGTC |
| *ank20*-452R | GCCTCTACATGATCCAAGACCACAGC |

*^1^F and R refer to primers that bind to the sense and antisense strand, respectively. The number immediately preceding F or R corresponds to the first nucleotide of the sequence that the primer targets.*

Table A2. Plasmids for cloning and expression of recombinant *O. tsutsugamushi* Anks

| Gene | pBMH^1^ | pEGFPC1^2^ | p3XFLAG-CMV-7.1^3^ | p3XFLAG-CMV-14^4^ |
| --- | --- | --- | --- | --- |
| *ank1_02* | pBMH-Ank1_02 | pEGFP-Ank1_02 | pFlag-Ank1_02 |  |
| *ank2* | pBMH-Ank2 | pEGFP-Ank2 | pFlag-Ank2 |  |
| *ank3_08* | pBMH-Ank3_08 | pEGFP-Ank3_08 | pFlag-Ank3_08 |  |
| *ank4_01* | pBMH-Ank4_01 | pEGFP-Ank4_01 | pFlag-Ank4_01 | pAnk4-Flag_01 |
| *ank5_01* | pBMH-Ank5_01 | pEGFP-Ank5_01 | pFlag-Ank5_01 |  |
| *ank6_02* | pBMH-Ank6_02 | pEGFP-Ank6_02 | pFlag-Ank6_02 |  |
| *ank7_02* | pBMH-Ank7_02 | pEGFP-Ank7_02 | pFlag-Ank7_02 |  |
| *ank8* | pBMH-Ank8 | pEGFP-Ank8 | pFlag-Ank8 |  |
| *ank9* | pBMH-Ank9 | pEGFP-Ank9 | pFlag-Ank9 | pAnk9-Flag |
| *ank10_01* | pBMH-Ank10_01 | pEGFP-Ank10_01 |  |  |
| *ank11* | pBMH-Ank11 | pEGFP-Ank11 | pFlag-Ank11 |  |
| *ank12_01* | pBMH-Ank12_01 | pEGFP-Ank12_01 | pFlag-Ank12_01 |  |
| *ank13* | pBMH-Ank13 | pEGFP-Ank13 | pFlag-Ank13 |  |
| *ank14* | pBMH-Ank14 | pEGFP-Ank14 |  |  |
| *ank15* | pBMH-Ank15 | pEGFP-Ank15 | pFlag-Ank15 |  |
| *ank16* | pBMH-Ank16 | pEGFP-Ank16 | pFlag-Ank16 |  |
| *ank17* | pBMH-Ank17 | pEGFP-Ank17 | pFlag-Ank17 | pAnk17-Flag |
| *ank18* | pBMH-Ank18 | pEGFP-Ank18 | pFlag-Ank18 |  |
| *ank19* | pBMH-Ank19 | pEGFP-Ank19 | pFlag-Ank19 |  |
| *ank20* | pBMH-Ank20 | pEGFP-Ank20 | pFlag-Ank20 |  |

*^1^pBMH constructs carried the ank genes that were codon-optimized for expression in mammalian cells and served as templates for cloning the codon-optimized genes into all other constructs used in this study with the exception of pEGFP-Ank10 and pEGFP-Ank14, as indicated in the Materials and Methods section.*

*^2^pEGFP constructs were used to express the codon-optimized Anks N-terminally fused to eGFP.*

*^3^p3xFlag7 constructs were used to express the Anks with triplicate Flag tags at their N-termini.*

*^4^p3xFlag14 constructs were used to express the Anks with triplicate Flag tags at their C-termini.*

Table A3. Oligonucleotides used for cloning *ank* genes into p3xFLAG-CMV-14

| Designation | Sequence (5’ to 3’)^1^ |
| --- | --- |
| *Kpn*I*-ank4-*1F | **TGCT**GGTACC*A*ATGAACAATGGGAACCTGCTG |
| *Xba*I-*ank4-*1176R | **GATC**TCTAGAGCTGTTGGTGTTCTCGCC |
| *Kpn*I*-ank9-*1F | **TGCT**GGTACC*A*ATGGGGAGATTCACCAGACTG |
| *Xba*I*-ank9-*1266R | **GATC**TCTAGAGTCGCAGATGGCGTAGC |
| *Kpn*I*-ank17-*1F | **TGCT**GGTACC*A*ATGAACAAACTGAACCAACTGG |
| *Xba*I*-ank17-*726R | **GATC**TCTAGAGGCGTTGTTGTCGTCGAT |

*^1^Bold nucleotides correspond to extra nucleotides upstream of KpnI or XbaI sites; underlined nucelotides correspond to restriction sites; italicized nucleotide corresponds to extra base added to restore the reading frame.*

Table A4. Oligonucleotides used for cloning HlyA and chimeric HlyA fusion coding sequences into pET19b

| Designation | Sequence (5’ to 3’)^1^ |
| --- | --- |
| pET19b-*hlyA*-4F | ACGACGACAAGCATATGCCAACAATAACCACTGCACAAA |
| pET19b-*hlyA*-3075R | GGATCCTCGAGCATATGTTATGCTGATGCTGTCAAAGTTATT |
| pET19b-*hlyA*-2892R | GGATCCTCGAGCATATGTTATGCATCATTCCCATACACA TAA CTT |
| *hlyA*-1731F | TAAATGGACGGTGAAGGGGGT |
| *hlyA-*2892-*ank1-*822R | **GGCATCCTGGTTGCT**^4^TGCATCATTCCCATACACATAACTT |
| *ank1-*808F | **AGCAACCAGGATGCCAAC** |
| pET19b*-ank1-*990R | GGATCCTCGAGCATATG**TTACTCTTCCTCGTAGATGGCG** |
| *hlyA-*2892R*-ank2-*867R | **GCTTTCGAAGATCTC**TGCATCATTCCCATACACATAACTT |
| *ank2-*853F | **GAGATCTTCGAAAGCAACCAC** |
| pET19b*-ank2-*1035R | GGATCCTCGAGCATATG**TTACTCGCCCTCGTACAG** |
| *hlyA-*2892R*-ank3-*540R | **GATGTCGGCGCCATA**TGCATCATTCCCATACACATAACTT |
| *ank3-*526F | **TATGGCGCCGACATCGAC** |
| pET19b*-ank3-702R* | GGATCCTCGAGCATATG**TTAACAGGAGGAGAACAGCTG** |
| *hlyA-*2892*-ank4-*1011R | **CGGCTTCAGGATCTG**TGCATCATTCCCATACACATAACTT |
| *ank4-*997F | **CAGATCCTGAAGCCGGAC** |
| pET19b*-ank4-*1179R | GGATCCTCGAGCATATG**TTAAGAGTTGGTGTTTTCACCTTTC** |
| *hlyA-*2892*-ank5-*870R | **CGCGTCTTGGTTAGA**TGCATCATTCCCATACACATAACTT |
| *ank5-*856F | **TCTAACCAAGACGCGAACC** |
| pET19b*-ank5-*2892R | GGATCCTCGAGCATATG**TTACTCGCCCTCGTAGATAGC** |
| *hlyA-*2892*-ank6-*846R | **TTGGGACTTCTGGCT**TGCATCATTCCCATACACATAACTT |
| *ank6-*832F | **AGCCAGAAGTCCCAAACC** |
| pET19b*-ank6-*1108R | GGATCCTCGAGCATATG**TTACTCCTCTTCGTATATTGCGTG** |
| *hlyA-*2892*-ank7-*1089R | **GATCTCGTCCTGGGT**TGCATCATTCCCATACACATAACTT |
| *ank7-*1075F | **ACCCAGGACGAGATCGC** |
| pET19b*-ank7-*1233R | GGATCCTCGAGCATATG**TTAGTTGTTTGTGTAATTTTGGGCC** |
| *hlyA-*2892*-ank8-*1002R | **GATGCTCTCGACGGC**TGCATCATTCCCATACACATAACTT |
| *ank8-*988F | **GCCGTCGAGAGCATCGAC** |
| pET19b*-ank8-*1170R | GGATCCTCGAGCATATG**TTAGCTTTCTTTGTGCATGACG** |
| *hlyA-*2892*-ank9-*1101R | **GACGGTCTCGGCCAG**TGCATCATTCCCATACACATAACTT |
| *ank9-*1087F | **CTGGCCGAGACCGTCACA** |
| pET19b*-ank9-*1269R | GGATCCTCGAGCATATG**TTAGTCGCAGATGGCGTAGCC** |
| *hlyA-*2892*-ank10* 1488R | **GCTCTTGTGGCTCTC**TGCATCATTCCCATACACATAACTT |
| *ank10-*1474F | **GAGAGCCACAAGAGCATTA** |
| pET19b*-ank10* 1656R | GGATCCTCGAGCATATG**TTAGGTCTCAATGTTCACCACC** |
| *hlyA-*2892*-ank11-*522R | **CATCCTGCAGATGCA**TGCATCATTCCCATACACATAACTT |
| *ank11-*508F | **TGCATCTGCAGGATGGCC** |
| pET19b*-ank11-*690R | GGATCCTCGAGCATATG**TTACAGGCCGATGTTCTTTC** |
| *hlyA-*2892*-ank12* 1320R | **TGGTTGTTCCTTGCA**TGCATCATTCCCATACACATAACTT |
| *ank12-*1306F | **TGCAAGGAACAACCAAAGG** |
| pET19b*-ank12* 1488R | GGATCCTCGAGCATATG**TTAGATGCACCTGTGGCC** |
| *hlyA-*2892*-ank13-*1308R | **GTCGTCGATAGATTT**TGCATCATTCCCATACACATAACTT |
| *ank13-*1294F | **AAATCTATCGACGACGTGTGC** |
| pET19b*-ank13 1*476R | GGATCCTCGAGCATATG**TTAGATGCCGAGACTTGAAG** |
| *hlyA-*2892*-ank14-*1089R | **CTCGTTAATGTTGGT**TGCATCATTCCCATACACATAACTT |
| *ank14-*1075F | **ACCAACATTAACGAGCAAGC** |
| pET19b*-ank14-*1257R | GGATCCTCGAGCATATG**TTAGTTCTCCCCCATGGC** |
| *hlyA-*2892*-ank15-*762R | **AATGTTGTCCTTCTC**TGCATCATTCCCATACACATAACTT |
| *ank15-*748F | **GAGAAGGACAACATTCAGCCAC** |
| pET19b*-ank15-930R* | GGATCCTCGAGCATATG**TTACTCATTATTGGTGGTGTTTTGGC** |
| *hlyA-*2892*-ank16-*633R | **CTCGCTGTCGGGCTT**TGCATCATTCCCATACACATAACTT |
| *ank16-*619F | **AAGCCCGACAGCGAGAAG** |
| pET19b*-ank16* 801R | GGATCCTCGAGCATATG**TTAGATCATGCCGTGGATGC** |
| *hlyA--*2892*-ank17-*561R | **CAGTTTGGATTCCTT**TGCATCATTCCCATACACATAACTT |
| *ank17-*547F | **AAGGAATCCAAACTGTTCGG** |
| pET19b*-ank17-*729R | GGATCCTCGAGCATATG**TTAGGCGTTGTTGTCGTCG** |
| *hlyA-*2892*-ank18-*51R | **GTAGGCCTTGTACAG**TGCATCATTCCCATACACATAACTT |
| *ank18-*37F | **CTGTACAAGGCCTACTTCAGC** |
| pET19b*-ank18-*219R | GGATCCTCGAGCATATG**TTAGATGATGTTGCAGCCGGTG** |
| *hlyA-*2892*-ank19-*327R | **CATGTCCGCGCCGTG**TGCATCATTCCCATACACATAACTT |
| *ank19-*313F | **CACGGCGCGGACATGAAC** |
| pET19b*-ank19* 495R | GGATCCTCGAGCATATG**TTAACACTTCACGTTGGCG** |
| *hlyA-*2892*-ank20-*1362R | **CGGCAGATACTGGAA**TGCATCATTCCCATACACATAACTT |
| *ank20-*1348F | **TTCCAGTATCTGCCGAACG** |
| pET19b*-ank20-*1530R | GGATCCTCGAGCATATG**TTACACGCTGCTCTCGTTT** |
| *hlyA*-2892-*ompA*-447R | **AGCCCTCTTTTTCCC**TGCATCATTCCCATACACAT |
| *ompA*-433F | **GGGAAAAAGAGGGCTGATG** |
| pET19b-*ompA*-615R | GGATCCTCGAGCATATG**TTATGCTATATTACTTTTAATAATT** |
| *hlyA-*2892*-lktA-*2664R | **GATTTTACCGTTACC**TGCATCATTCCCATACACATAACTT |
| *lktA-*2650F | **GGTAACGGTAAAATCACCCAGG** |
| pET19b*-lktA-*2862R | GGATCCTCGAGCATATG**TTACGCCGCACGCGCGAA** |
|  | |

*^1^Underlined nucleotides correspond to pET19b vector sequence; unformatted text corresponds to hlyA sequence; double underlined nucleotides correspond to an added stop codon; bold nucleotides correspond to ank gene coding sequence.*

Table A5. Plasmids used for T1SS analyses

| Gene | Plasmid | Purpose |
| --- | --- | --- |
| *E. coli* *hlyA* | pHlyA | T1SS positive control substrate |
| *E. coli* *hlyA* nucleotides 1-2892 | pHlyA∆965-1024^1^ | T1SS negative control substrate |
| *E. coli* *hlyBD* | pLG575^2^ | Used to reconstitute the fully functional T1SS in *E. coli* BL21 (DE3) |
| *hlyA-lktA* | pHlyA-LktA^3^ | T1SS chimeric substrate positive control |
| *hlyA-ank1_02* | pHlyA-Ank1_02^4^ | Evaluate Ank1_02 C-terminus for T1SS |
| *hlyA-ank2* | pHlyA-Ank2 | Evaluate Ank2 C-terminus for T1SS |
| *hlyA-ank3_08* | pHlyA-Ank3_08 | Evaluate Ank3_08 C-terminus for T1SS |
| *hlyA-ank4_01* | pHlyA-Ank4_01 | Evaluate Ank4_01 C-terminus for T1SS |
| *hlyA-ank5_01* | pHlyA-Ank5_01 | Evaluate Ank5_01 C-terminus for T1SS |
| *hlyA-ank6_02* | pHlyA-Ank6_02 | Evaluate Ank6_02 C-terminus for T1SS |
| *hlyA-ank7_02* | pHlyA-Ank7_02 | Evaluate Ank7_02 C-terminus for T1SS |
| *hlyA-ank8* | pHlyA-Ank8 | Evaluate Ank8 C-terminus for T1SS |
| *hlyA-ank9* | pHlyA-Ank9 | Evaluate Ank9 C-terminus for T1SS |
| *hlyA-ank10_01* | pHlyA-Ank10_01 | Evaluate Ank10_01 C-terminus for T1SS |
| *hlyA-ank11* | pHlyA-Ank11 | Evaluate Ank11 C-terminus for T1SS |
| *hlyA-ank12_01* | pHlyA-Ank12_01 | Evaluate Ank12_01 C-terminus for T1SS |
| *hlyA-ank13* | pHlyA-Ank13 | Evaluate Ank13 C-terminus for T1SS |
| *hlyA-ank14* | pHlyA-Ank14 | Evaluate Ank14 C-terminus for T1SS |
| *hlyA-ank15* | pHlyA-Ank15 | Evaluate Ank15 C-terminus for T1SS |
| *hlyA-ank16* | pHlyA-Ank16 | Evaluate Ank16 C-terminus for T1SS |
| *hlyA-ank17* | pHlyA-Ank17 | Evaluate Ank17 C-terminus for T1SS |
| *hlyA-ank18* | pHlyA-Ank18 | Evaluate Ank18 C-terminus for T1SS |
| *hlyA-ank19* | pHlyA-Ank19 | Evaluate Ank19 C-terminus for T1SS |
| *hlyA-ank20* | pHlyA-Ank20 | Evaluate Ank20 C-terminus for T1SS |
| *hlyA-ompA* | pHlyA-OmpA | Evaluate OmpA C-terminus for T1SS |

*^1^HlyA∆965-1024 lacks its final C-terminal 60 amino acids that contain the type 1 secretion signal.*

*^2^Described in Mackman et al., 1985.*

*^3^HlyA-LktA is a chimeric protein consisting of HlyA lacking its final C-terminal 60 amino acids fused to the final 70 amino acids of LktA, which contains the LktA type 1 secretion signal.*

*^4^Each HlyA-Ank or HlyA-OmpA protein is a chimeric protein consisting of HlyA∆965-1024 fused to the final 60 amino acids of each O. tsutsugamushi Ank or OmpA, respectively.*

Table A6. Oligonucleotides used for cloning Ank coding sequences into pJB-CAT-CyaA

| Designation | Sequence (5’ to 3’)^1^ |
| --- | --- |
| cyaA-ank1_02-4F | **TTCCGGCTAT***GTCGAC*ATGAACAACTACCTGAGACTGAG |
| cyaA-ank1_02-990R | **GCATGCCTCAGTCGACTCA**CTCTTCCTCGTAGATGGCGTAGGC |
| cyaA-ank2-4F | **TTCCGGCTAT***GTCGAC*ATGGGCAACACAGCACTGCATGAG |
| cyaA-ank2-1035R | **GCATGCCTCAGTCGACTCA**CTCGCCCTCGTACAGGGCG |
| cyaA-ank3_08-4F | **TTCCGGCTAT***GTCGAC*ATGATCCTGAAGAGCAAGACCAAATAC |
| cyaA-ank3_08-708R | **GCATGCCTCAGTCGACTCA**ACAGGAGGAGAACAGCTGAT GG |
| cyaA-ank4-4F | **TTCCGGCTAT***GTCGAC*ATGAACAACGGTAACCTGCTGCACG |
| cyaA-ank4-1179R | **GCATGCCTCAGTCGACTCA**AGAGTTGGTGTTTTCACCTTTCAGTG |
| cyaA-ank5-4F | **TTCCGGCTAT***GTCGAC*ATGATGACAGTGCTTCACAAGGCCGCG |
| cyaA-ank5-1038R | **GCATGCCTCAGTCGACTCA**CTCGCCCTCGTAGATAGCGTAGG |
| cyaA-ank6_02-4F | **TTCCGGCTAT**G*TCGAC*ATGTACAAGGTTCTGCCACTCAGAG |
| cyaA-ank6_02-1011R | **GCATGCCTCAGTCGACTCA**CTCCTCTTCGTATATTGCGTGG |
| cyaA-ank7_02-4F | **TTCCGGCTAT***GTCGAC*ATGGATAAGACCCTGAGGCAGCAAG |
| cyaA-ank7_02-1257R | **GCATGCCTCAGTCGACTCA**GTTGTTTGTGTAATTTTGGGCCAGC |
| cyaA-ank8-4F | **TTCCGGCTAT***GTCGAC*ATGTATAATACCGACCTGCATGATG |
| cyaA-ank8-1170R | **GCATGCCTCAGTCGACTCA**GCTTTCTTTGTGCATGACGTTTCCC |
| cyaA-ank9-4F | **TTCCGGCTAT***GTCGAC*ATGGGGAGATTCACCAGACTGC |
| cyaA-ank9-1269R | **GCATGCCTCAGTCGACTCA**GTCGCAGATGGCGTAGCC |
| cyaA-ank10_01-4F | **TTCCGGCTAT***GTCGAC*ATGAAGAACTGGCTGAGCTGGC |
| cyaA-ank10_01-1656R | **GCATGCCTCAGTCGACTCA**GGTCTCAATGTTCACCACCTCC |
| cyaA-ank11-4F | **TTCCGGCTAT***GTCGAC*ATGAAGTCCAATAAGAGCCCCAGG |
| cyaA-ank11-687R | **GCATGCCTCAGTCGACTCA**CAGGCCGATGTTCTTTCCGC |
| cyaA-ank12_01-4F | **TTCCGGCTAT***GTCGAC*ATGATGAATACCGCTTTGTCACTGG |
| cyaA-ank12_01-1485R | **GCATGCCTCAGTCGACTCA**GATGCACCTGTGGCCGCTG |
| cyaA-ank13-4F | **TTCCGGCTAT***GTCGAC*ATGGCCCCTTTCTCTACCAAAGACG |
| cyaA-ank13-1473R | **GCATGCCTCAGTCGACTCA**GATGCCGAGACTTGAAGATTTATTC |
| cyaA-ank14-4F | **TTCCGGCTAT**GTC*GAC*ATGAACTTCCAACTGCATAACGC |
| cyaA-ank14-1257R | **GCATGCCTCAGTCGACTCA**GTTCTCCCCCATGGCATGC |
| cyaA-ank15-4F | **TTCCGGCTAT***GTCGAC*ATGAGCAGAGAGGAACTGCTGC |
| cyaA-ank15-930R | **GCATGCCTCATCGACTCA**CTCATTATTGGTGGTGTTTTGGC |
| cyaA-ank16-4F | **TTCCGGCTAT**G*TCGAC*ATGAATAACAACGACCTGCTGAG |
| cyaA-ank16-801R | **GCATGCCTCAGTCGACTCA**GATCATGCCGTGGATGCTG |
| cyaA-ank17-4F | **TTCCGGCTAT***GTCGAC*ATGAACAAACTGAACCAACTGGATAAG |
| cyaA-ank17-729R | **GCATGCCTCAGTCGACTCA**GGCGTTGTTGTCGTCGATG |
| cyaA-ank18-4F | **TTCCGGCTAT***GTCGAC*ATGGACGACGCCACCAGC |
| cyaA-ank18-219R | **GCATGCCTCAGTCGACTCA**GATGATGTTGCAGCCGGTG |
| cyaA-ank19-4F | **TTCCGGCTAT***GTCGAC*ATGAGGAACCCCTTCGCTTACTGC |
| cyaA-ank19-492R | **GCATGCCTCAGTCGACTCA**ACACTTCACGTTGGCGCCC |
| cyaA-ank20-4F | **TTCCGGCTAT***GTCGA*CATGGACCAGCTGCTGAGGTATATC |
| cyaA-ank20-1527R | **GCATGCCTCAGTCGACTCA**CACGCTGCTCTCGTTTCCC |

*^1^Bold nucleotides correspond to pJB-CAT-CyaA vector sequence; italicized nucleotides correspond to SalI restriction site; underlined nucleotides correspond to added start codon.*

Table A7. Plasmids used for T4SS analyses

| Gene | Plasmid | Purpose |
| --- | --- | --- |
| *cyaA-ank1_02* | pCyaA-Ank1_02 | Evaluate Ank1_02 for T4SS |
| *cyaA -ank2* | pCyaA-Ank2 | Evaluate Ank2 for T4SS |
| *cyaA -ank3_08* | pCyaA-Ank3_08 | Evaluate Ank3_08 for T4SS |
| *cyaA -ank4_01* | pCyaA-Ank4_01 | Evaluate Ank4_01 for T4SS |
| *cyaA -ank5_01* | pCyaA-Ank5_01 | Evaluate Ank5_01 for T4SS |
| *cyaA -ank6_02* | pCyaA-Ank6_02 | Evaluate Ank6_02 for T4SS |
| *cyaA -ank7_02* | pCyaA-Ank7_02 | Evaluate Ank7_02 for T4SS |
| *cyaA -ank8* | pCyaA-Ank8 | Evaluate Ank8 for T4SS |
| *cyaA -ank9* | pCyaA-Ank9 | Evaluate Ank9 for T4SS |
| *cyaA -ank10_01* | pCyaA-Ank10_01 | Evaluate Ank10_01 for T4SS |
| *cyaA -ank11* | pCyaA-Ank11 | Evaluate Ank11 for T4SS |
| *cyaA -ank12_01* | pCyaA-Ank12_01 | Evaluate Ank12_01 for T4SS |
| *cyaA -ank13* | pCyaA-Ank13 | Evaluate Ank13 for T4SS |
| *cyaA -ank14* | pCyaA-Ank14 | Evaluate Ank14 for T4SS |
| *cyaA -ank15* | pCyaA-Ank15 | Evaluate Ank15 for T4SS |
| *cyaA -ank16* | pCyaA-Ank16 | Evaluate Ank16 for T4SS |
| *cyaA -ank17* | pCyaA-Ank17 | Evaluate Ank17 for T4SS |
| *cyaA -ank18* | pCyaA-Ank18 | Evaluate Ank18 for T4SS |
| *cyaA -ank19* | pCyaA-Ank19 | Evaluate Ank19 for T4SS |
| *cyaA -ank20* | pCyaA-Ank20 | Evaluate Ank20 for T4SS |

Table A8. GFP-Ank colocalization with ER markers

| Protein | ER lumen^1^ | ER membrane^2^ | Ank staining pattern |
| --- | --- | --- | --- |
| GFP | - | - | Diffuse cytosolic |
| GFP-Ank1_02 | ++ | ++ | Perinuclear and cytosolic aggregative |
| GFP-Ank2 | ++ | - | Perinuclear, aggregative |
| GFP-Ank3_08 | + | - | Perinuclear and cytosolic aggregative |
| GFP-Ank4_01 | ++ | ++ | Perinuclear, aggregative |
| GFP-Ank5_01 | + | ++ | Perinuclear and cytosolic aggregative |
| GFP-Ank6_02 | - | - | Perinuclear and cytosolic aggregative |
| GFP-Ank7_02 | - | - | Perinuclear and cytosolic aggregative |
| GFP-Ank8 | - | - | Perinuclear and cytosolic aggregative |
| Flag-Ank8 | + | - | Perinuclear and cytosolic aggregative |
| GFP-Ank9 | +++ | +++ | Perinuclear and cytosolic aggregative |
| GFP-Ank10_01 | + | - | Perinuclear and cytosolic aggregative |
| GFP-Ank11 | ++ | - | Reticulate |
| GFP-Ank12_01 | - | - | Diffuse cytosolic |
| GFP-Ank13 | - | - | Primarily nuclear, diffuse cytosolic |
| GFP-Ank14 | - | - | Perinuclear and cytosolic aggregative |
| GFP-Ank15 | - | - | Perinuclear and cytosolic aggregative |
| Flag-Ank15 | + | - | Perinuclear and cytosolic aggregative |
| GFP-Ank16 | +++ | +++ | Perinuclear and cytosolic aggregative |
| GFP-Ank17 | - | - | Diffuse cytosolic |
| GFP-Ank18 | - | + | Perinuclear and cytosolic aggregative |
| GFP-Ank19 | - | + | Perinuclear and cytosolic aggregative |
| GFP-Ank20 | + | + | Perinuclear and cytosolic aggregative |

^1^The ER lumen was stained with calreticulin for all GFP-Anks. Flag-Ank8 and Flag-Ank15 colocalized with ER lumenal marker, protein disulfide isomerase.

^2^The ER membrane was stained with calnexin for all Anks.
